# Transferosomes as Drug Delivery Systems: Design Principles, Deformability, and Translational Challenges

**DOI:** 10.3390/ph19060956

**Published:** 2026-06-19

**Authors:** Enrique A. Nieves, María C. Cotto, Francisco Márquez

**Affiliations:** 1 Department of Pharmaceutical Sciences, Nova Southeastern University, Puerto Rico Campus, San Juan, PR 00926, USA; nenrique@nova.edu; 2 Nanomaterials Research Group, School of Natural Sciences and Technology, Division of Natural Sciences, Technology and Environment, Universidad Ana G. Méndez-Gurabo Campus, Gurabo, PR 00778, USA; mcotto48@uagm.edu

**Keywords:** transferosomes, ultradeformable vesicles, liposomes, edge activators, deformability, drug delivery, transdermal delivery, biological barriers, critical quality attributes, translational challenges

## Abstract

Transferosomes are liposome-derived ultradeformable vesicles designed to improve drug delivery across restrictive biological barriers, particularly in non-invasive administration routes. Their structure is based on phospholipid bilayers modified with edge activators, usually surfactants or bile salts, which increase membrane flexibility while preserving vesicular organization. This balance between deformability and stability distinguishes transferosomes from conventional liposomes and has supported their use in dermal, transdermal, ocular, nasal, buccal, and other mucosal delivery systems. However, despite extensive experimental interest, the field remains limited by inconsistent terminology, heterogeneous formulation strategies, non-harmonized deformability assays, and incomplete translation from laboratory formulations to clinically relevant products. This review critically examines transferosomes from a formulation-development perspective, focusing on the relationship between lipid composition, edge-activator selection, vesicle properties, deformability, drug release, and biological performance. Particular attention is given to critical quality attributes, analytical characterization, mechanistic interpretations of barrier interaction, and the unresolved debate between intact vesicle penetration, drug-release-dominated delivery, and barrier perturbation. Transferosomes are also positioned in comparison with conventional liposomes, ethosomes, and transethosomes. Finally, the review identifies key unmet needs related to standardization, reproducibility, scalability, storage stability, and regulatory uncertainty. By integrating formulation design with mechanistic and translational analysis, this review aims to clarify when transferosomes offer a genuine delivery advantage and which parameters must be controlled to support their further pharmaceutical development.

## 1. Introduction

Biological barriers are essential for maintaining physiological homeostasis, protecting tissues from pathogens and xenobiotics, and regulating molecular transport between compartments. At the same time, these barriers represent one of the main obstacles to effective pharmacotherapy because they frequently prevent therapeutic agents from reaching target tissues at therapeutically relevant concentrations [1,2]. Consequently, many active compounds show limited in vivo performance not because of insufficient pharmacological activity, but because of poor absorption, restricted tissue penetration, rapid clearance, or limited intracellular access [1,2,3]. For this reason, advanced drug delivery systems have become central tools to improve drug localization, increase bioavailability, reduce systemic toxicity, and overcome physiological transport constraints [1,2].

These challenges are route- and tissue-dependent. Therapeutic agents may encounter epithelial and endothelial barriers, mucus layers, extracellular matrices, cellular membranes, intracellular trafficking constraints, and highly specialized interfaces such as the blood–brain barrier (BBB) [1,4]. In practice, delivery may be limited by the stratum corneum, gastrointestinal mucus and epithelium, ocular surface, nasal mucosa, or BBB, each of which imposes distinct physicochemical and biological restrictions on transport [1,4,5]. These sequential limitations are particularly relevant for poorly soluble drugs, high-molecular-weight compounds, peptides, proteins, and nucleic-acid-based therapeutics, whose clinical performance is often dictated more by delivery efficiency than by intrinsic potency [2,3].

Among peripheral barriers, the skin remains one of the most effective and widely studied. Its outermost layer, the stratum corneum, is a highly organized lipid-rich structure that severely limits the permeation of most hydrophilic molecules and macromolecules [1,5]. Although transdermal administration offers clear advantages, including avoidance of gastrointestinal degradation and first-pass hepatic metabolism, only a limited number of drugs can be effectively delivered through conventional passive systems because of the stringent barrier properties of the skin [1,5]. Mucosal barriers also present important constraints, since mucus can hinder drug and nanoparticle diffusion through steric obstruction, adhesive interactions, hydrophobic domains, and electrostatic effects [6,7,8]. In the gastrointestinal tract, these constraints are compounded by enzymatic degradation, luminal fluid dynamics, pH gradients, epithelial tight junctions, and presystemic metabolism [6,7,8]. Likewise, the BBB tightly regulates exchange between the bloodstream and the central nervous system through specialized endothelial cells, tight junctions, supporting cells, and transporter systems, thereby excluding most circulating drugs and making central nervous system delivery especially demanding [9,10]. This brief overview illustrates why biological barriers remain a central obstacle in drug delivery, without attempting to provide an exhaustive description of each barrier.

These limitations have driven the emergence of advanced drug delivery systems specifically engineered to navigate biological barriers more effectively than free drug molecules. Such systems include nanoparticles, vesicular carriers, ligand-targeted constructs, stimuli-responsive materials, and physical enhancement technologies designed to improve tissue penetration, prolong residence time, protect labile drug molecules, and increase delivery specificity [1,4]. Their purpose is not only to transport drugs across restrictive barriers, but also to modulate the interaction between the formulation and the biological interface in a controlled and predictable manner [1,2]. Within this broader context, deformable vesicular carriers have attracted particular interest because they combine encapsulation capacity with tunable interfacial properties and enhanced adaptability to confined biological environments. This conceptual framework explains the growing relevance of transferosomes and other ultradeformable vesicles as promising platforms for non-invasive and minimally invasive drug delivery [1,4].

Transferosomes are phospholipid-based vesicular carriers specifically designed to combine the structural advantages of liposomes with a markedly enhanced ability to deform under stress. In their classical form, they consist of a phospholipid bilayer incorporating one or more edge activators, typically single-chain surfactants or bile salts, which partially destabilize lipid packing and increase membrane flexibility without causing complete vesicle disintegration [11,12,13]. Therefore, transferosomes can be practically understood as liposome-derived ultradeformable vesicles in which the bilayer is intentionally modified with edge activators to increase membrane flexibility while preserving vesicular organization. This structural concept distinguishes transferosomes from conventional liposomes and underpins their widespread use as ultradeformable carriers for topical and transdermal drug delivery [12,14].

The relevance of transferosomes in non-invasive delivery lies in their capacity to adapt their shape to confined biological environments while maintaining vesicular integrity to a greater extent than rigid or less deformable lipid systems. This behavior has been used to explain their improved performance in transporting active compounds into or across the skin, especially under non-occlusive conditions, where a transepidermal hydration gradient has been proposed as an additional driving force for vesicle movement [13,15]. Although the precise contribution of intact vesicle penetration versus localized drug release remains debated, there is broad agreement that transferosomal formulations can enhance drug deposition in deeper skin layers and, in some cases, improve transdermal flux compared with conventional liposomes or simple drug solutions [12,16].

A key reason for the continuing interest in transferosomes is their compositional versatility. By modulating phospholipid type, edge activator identity, surfactant concentration, aqueous phase composition, and post-processing conditions, it is possible to tune vesicle size, lamellarity, drug loading, colloidal stability, and deformability over a broad range [11,12,14]. This adaptability allows transferosomes to accommodate both hydrophilic and lipophilic compounds and has encouraged their evaluation for the delivery of small molecules, peptides, proteins, phytochemicals, and other poorly permeable actives [12,14]. Their performance, however, should not be reduced to a generic claim of enhanced penetration. Comparative studies with related ultradeformable vesicles, including ethosomes and transethosomes, show that delivery outcomes depend on the interplay between vesicle composition, drug physicochemical properties, deformability, and the biological model employed [15]. Beyond skin delivery, transferosomes have also attracted interest for other non-invasive or minimally invasive routes, including nasal, ocular, buccal, and mucosal delivery, where deformability, interfacial adaptability, and encapsulation capacity may also be advantageous [17]. Nevertheless, their scientific identity remains closely linked to their original role as ultradeformable vesicles developed to negotiate restrictive barriers more efficiently than conventional lipid carriers [13].

At the same time, the growing literature on transferosomes has revealed persistent conceptual and methodological issues. The term is sometimes used loosely for formulations that differ substantially in composition or mechanism from the original concept, and claims regarding superior penetration are not always supported by harmonized characterization data or adequately controlled comparative studies [15,16]. These limitations justify a more critical examination of transferosomes not only as promising nanocarriers, but also as formulation-dependent systems whose true potential depends on rigorous design, meaningful deformability assessment, appropriate comparators, and careful interpretation of biological performance.

Several reviews have already established the conceptual basis of transferosomes and documented their value as deformable lipid carriers, especially for transdermal delivery. Earlier contributions clarified the theoretical framework of highly deformable vesicles, the role of membrane flexibility in transport, and the distinction between transferosomes and related carrier systems [11,18]. More recent reviews have summarized transferosome composition, preparation methods, characterization techniques, and transdermal applications, while also highlighting the growing relevance of quality-by-design (QbD) principles in formulation development [12,17]. In parallel, comparative studies and broader discussions on ultradeformable vesicles have expanded the field beyond transferosomes alone by placing them alongside ethosomes and transethosomes [15,19]. Taken together, this literature provides a solid foundation, but it also shows that previous reviews have mainly emphasized composition, preparation, characterization, and applications, whereas the current field requires a more explicit integration of formulation design, measurable quality attributes, deformability assessment, mechanistic interpretation, and translational readiness [12,15,19].

The present review is therefore not intended to duplicate earlier summaries of transferosome composition or transdermal applications. Its intended contribution is to reassess transferosomes as formulation-dependent pharmaceutical systems whose performance must be interpreted through the combined analysis of composition, process variables, vesicle architecture, deformability, release behavior, barrier interaction, and development constraints. This perspective is timely because the field has evolved in ways that are not fully captured by the existing literature. First, much of the influential review literature remains centered on transferosomes as transdermal systems, whereas recent primary research shows a broader application space that now includes ocular delivery and more sophisticated topical therapeutic designs [17,20,21]. Second, the literature has become increasingly formulation-driven: newer studies frequently employ experimental design, statistical optimization, and multi-response QbD strategies to tune vesicle performance, reflecting a shift from descriptive formulation development toward more systematic pharmaceutical engineering [12,20,22]. Third, the transferosome field now coexists with a larger family of deformable vesicles, making it more important to define what is specific to transferosomes and what is shared across related systems [15,19]. These developments create a need for an updated synthesis that is not limited to applications, but instead examines transferosomes through the lens of design principles, measurable quality attributes, and translational relevance.

Several core scientific questions remain insufficiently resolved. Although deformability is widely presented as the defining functional feature of transferosomes, its experimental assessment is still far from harmonized, and the relationship between deformability measurements and biological performance is often assumed rather than critically demonstrated [11,12,18]. Likewise, the literature does not consistently integrate composition, processing variables, vesicle architecture, and delivery outcomes into a single framework. As a result, studies often report size, polydispersity, zeta potential, entrapment efficiency, and permeation results without clearly establishing which parameters are truly predictive of performance and which merely describe the formulation [12,17,18]. The need for a more rigorous quality-oriented interpretation is further underscored by the increasing use of QbD-based optimization strategies in recent transferosome studies, which implicitly recognize that critical material attributes and critical process parameters must be linked to functional outcomes if formulations are to become reproducible and scalable [12,20,22].

The translational dimension of transferosome research also remains underdeveloped. Existing articles typically summarize formulation methods and biological applications, but they less frequently examine in a systematic way the issues that determine whether a promising laboratory formulation can become a robust pharmaceutical product. These issues include batch-to-batch reproducibility, long-term storage stability, industrially relevant manufacturing methods, meaningful comparators, and the minimum characterization package needed to support inter-study comparability [12,17]. This gap is particularly relevant because transferosomes are often described as highly promising carriers, yet the literature still lacks a broadly adopted framework for reporting, benchmarking, and interpreting their performance across studies [12,17,19]. A review that explicitly connects transferosome design with critical quality attributes, deformability assessment, mechanistic interpretation, and translational constraints can therefore offer added value beyond existing narrative summaries.

## 2. Structural Basis and Formulation Design of Transferosomes

The performance of transferosomes as drug delivery systems is fundamentally determined by the relationship between their structural organization and formulation design. Unlike conventional vesicular carriers, transferosomes are not defined solely by the presence of a lipid bilayer, but by a composition intentionally engineered to generate membrane flexibility, adaptive behavior under mechanical stress, and adequate drug-loading capacity [11,12,19,23]. Their pharmaceutical behavior must therefore be analyzed as the result of interconnected variables, including phospholipid composition, the nature and proportion of edge activators, the presence of auxiliary excipients, and the preparation and post-processing methods used during formulation [11,12,19].

This section examines the structural basis of transferosomes and the formulation factors that govern their physicochemical and functional properties. The discussion focuses on how membrane composition, auxiliary components, drug-related variables, and process conditions determine vesicle attributes such as deformability, colloidal stability, encapsulation behavior, and delivery performance [11,12,19,23]. This integrated perspective is essential because, in transferosomal systems, structure and formulation are inseparable determinants of function rather than independent formulation descriptors [12,23].

### 2.1. Defining Features of Transferosomes

Transferosomes are generally described as phospholipid-based, liposome-derived ultradeformable vesicular carriers in which membrane flexibility is intentionally increased through the incorporation of one or more edge activators into the bilayer [11,18,23,24]. In contrast to conventional liposomes, whose bilayers are comparatively more ordered and less adaptable under mechanical stress, transferosomes are designed to tolerate substantial shape deformation while preserving vesicular continuity to a functionally relevant extent [11,18]. This feature explains why the concept of transferosomes has historically been associated with enhanced transport across narrow intercellular pathways, especially in the context of non-occlusive topical or transdermal application [23,24].

The defining structural basis of a transferosome lies in the coexistence of two elements: a phospholipid bilayer that provides vesicular organization and drug compartmentalization, and an edge activator that partially destabilizes lipid packing and lowers the energetic cost of membrane deformation [11,18,19]. Edge activators are typically single-chain surfactants or bile salts with a relatively high curvature tendency, and their presence promotes local bilayer flexibility by introducing packing defects and dynamic heterogeneity into the membrane [11,18]. Importantly, the transferosome concept depends on a controlled balance between structural cohesion and membrane softness, so that the carrier remains sufficiently stable for drug loading and dispersion, yet sufficiently deformable to respond to external stress [11,19]. From a formulation standpoint, this balance distinguishes transferosomes from both rigid liposomes and excessively fluid systems that may lose colloidal or encapsulation stability [11,18].

A practical definition of transferosomes, therefore, requires more than stating that a formulation contains phospholipids and a surfactant. At minimum, the system should exhibit vesicular organization, include a membrane-softening component that acts as an edge activator, and show evidence of deformability beyond that expected for conventional liposomes prepared under comparable conditions [11,18,23]. In this sense, transferosomes are best understood as a functional subclass of deformable lipid vesicles rather than as a purely compositional category, particularly because the borders between transferosomes, elastic liposomes, ultradeformable vesicles, and related platforms are sometimes blurred [11,18,23].

**Figure 1:**
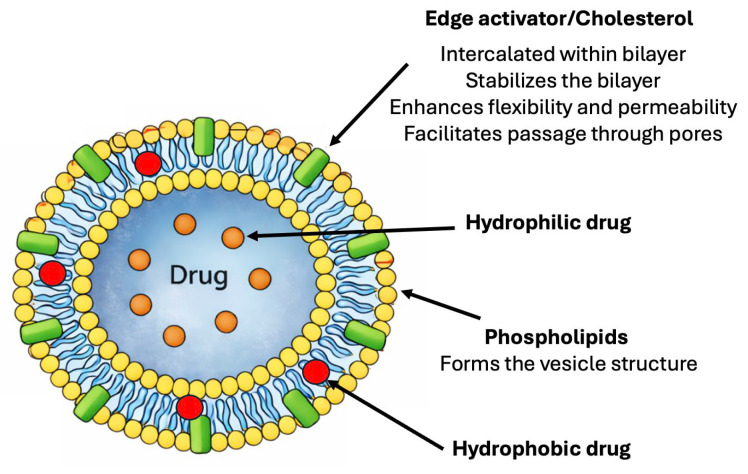
Schematic structure of a transferosome and the role of each component. Transferosomes are vesicular systems composed of a phospholipid bilayer incorporating edge activators (surfactants) and, optionally, cholesterol, both intercalated within the bilayer. Edge activators increase membrane flexibility and permeability by disrupting lipid packing, thereby enhancing vesicle deformability and facilitating passage through narrow intercellular pores. In contrast, cholesterol contributes to membrane stabilization and modulates bilayer fluidity. Hydrophilic drugs are encapsulated in the aqueous core, whereas hydrophobic drugs are preferentially located within the lipid bilayer.

Taken together, transferosomes are best defined through the combined consideration of composition, structure, and function. Their identity is rooted in phospholipid self-assembly, modulated by edge activators, and expressed through enhanced membrane deformability [11,12,18,23,24]. This working definition provides the basis for analyzing formulation variables, characterization strategies, and the extent to which deformability can translate into meaningful pharmaceutical performance. Figure 1 provides a schematic overview of a transferosome with its different components.

### 2.2. Role of Phospholipids and Edge Activators

The functional behavior of transferosomes is largely determined by the interplay between phospholipids and edge activators, since these two components define the organization, elasticity, and stability of the vesicular membrane [11,12,18,19]. While phospholipids provide the structural framework required for bilayer formation and drug compartmentalization, edge activators modulate intermolecular packing within the membrane and reduce its resistance to deformation [11,18]. In practical terms, transferosomes can therefore be viewed as formulation systems in which phospholipid self-assembly is deliberately tuned by amphiphilic additives to produce a membrane that is softer and more stress-responsive than that of conventional liposomes [12,18,19].

Phospholipids are the primary constituents of the transferosomal bilayer and play a decisive role in vesicle formation, drug incorporation, and membrane cohesion [11,12]. In most reported formulations, phosphatidylcholine-rich materials are preferred because they readily form bilayers, are generally biocompatible, and are well tolerated in topical applications [12,19]. Their amphiphilic structure allows the formation of closed vesicles with an aqueous core and a hydrophobic bilayer domain, thereby enabling the simultaneous incorporation of hydrophilic and lipophilic compounds [11,12]. However, phospholipids should not be regarded as interchangeable materials. Their acyl chain length, degree of unsaturation, purity, and phase transition temperature can significantly influence bilayer fluidity, packing density, permeability, and susceptibility to oxidation or hydrolysis [11,18]. As a result, the selection of a phospholipid source and composition may affect not only deformability but also vesicle stability, drug retention, and reproducibility.

Membrane behavior is further shaped by the amount of phospholipid present relative to the other formulation components. A sufficiently high phospholipid fraction is required to preserve bilayer continuity and vesicular integrity, whereas excessive membrane perturbation may shift the system toward mixed micelles, leaky vesicles, or poorly reproducible colloidal dispersions [12,23]. This is particularly relevant in transferosome design because the characteristic softness of the vesicle depends on controlled bilayer destabilization rather than on complete structural disruption. In this sense, phospholipids act not only as structural components but also as modulators of the formulation window within which deformability can be achieved without compromising pharmaceutical utility [11,12,23].

Edge activators are the second defining component of transferosomes and are responsible for generating the membrane flexibility that distinguishes these vesicles from conventional liposomes [11,12,18,19]. They are generally surfactants or bile salts that intercalate into the phospholipid bilayer, disrupt local lipid packing, and lower the energy required for bilayer bending and shape adaptation [11,18]. Common examples include Tween 80, Span 80, sodium cholate, and sodium deoxycholate, although other surfactants have also been employed depending on the intended application and compatibility with the drug cargo [12,19,23,25]. The mechanistic role of these molecules is not simply to fluidize the membrane in a generic sense, but to create transient packing defects and lateral heterogeneity that facilitate deformation under stress while retaining the vesicular character of the system [11,18].

The nature of the edge activator strongly affects transferosome performance. Non-ionic surfactants such as Tween 80 and Span 80 are frequently used because of their formulation versatility and broad pharmaceutical compatibility, whereas bile salts can produce marked membrane softening but may also alter vesicle stability and drug retention in different ways [12,19,23,25]. Their hydrophilic–lipophilic balance, chain structure, and affinity for the phospholipid domain influence how they partition into the bilayer and how strongly they perturb membrane organization [18,23]. Consequently, two transferosomal formulations containing the same phospholipid but different edge activators may show substantial differences in size, entrapment efficiency, elasticity, leakage, and skin permeation behavior [23,25]. This point is critical because it underscores that deformability is not an intrinsic property of all transferosome-like systems, but the outcome of a specific compositional balance.

The concentration of the edge activator is equally important. At low to moderate levels, it generally enhances membrane flexibility and may improve deformability and permeation performance; however, beyond an optimal range, increasing the surfactant fraction can destabilize the bilayer, reduce entrapment efficiency, promote drug leakage, or even impair vesicle formation [12,23,25]. This non-linear behavior has been reported in multiple formulation studies and is one of the main reasons why optimization of phospholipid-to-edge-activator ratio is considered a critical step in transferosome development [12,23]. In other words, the beneficial effect of edge activators is conditional rather than absolute: the same component that confers elasticity can also become a source of instability if used at an inappropriate concentration or in an unsuitable lipid environment [11,12,25].

The role of phospholipids and edge activators should therefore be interpreted as inherently interdependent. Phospholipids define the basic architecture and cohesion of the vesicle, whereas edge activators modulate the mechanical response of that architecture under stress [11,12,18,19]. Neither component alone explains transferosomal behavior. A phospholipid bilayer without a membrane-softening additive will behave more like a conventional liposome, whereas an excess of surfactant relative to phospholipid may lead to systems that no longer retain the structural and functional features expected of transferosomes [12,23]. This interdependence is central to rational formulation design and provides the basis for understanding why transferosome optimization must focus not only on composition itself, but also on how that composition translates into measurable membrane properties.

Overall, phospholipids and edge activators form the compositional core of transferosomes, and their balance governs the transition from a conventional vesicle to a genuinely deformable carrier [11,12,18,19,23,25]. Understanding their individual and combined roles is therefore essential for interpreting subsequent aspects of formulation behavior, including colloidal stability, drug loading, deformability measurements, and barrier interaction.

### 2.3. Influence of Auxiliary Components on Vesicle Behavior

Beyond phospholipids and edge activators, transferosomal performance is also shaped by a series of auxiliary components that, although not always considered defining ingredients, can significantly influence vesicle organization, colloidal stability, surface charge, drug retention, storage behavior, and ultimately delivery performance [11,12,19,26]. These components include cholesterol, charge-inducing agents, hydration media, and stabilizing excipients used during processing or long-term storage. Their contribution is particularly important because transferosomes operate within a narrow balance between membrane flexibility and structural integrity; therefore, relatively small compositional changes may alter vesicle behavior in ways that are not apparent from the primary lipid–surfactant composition alone [11,12,19].

One of the most frequently discussed auxiliary components is cholesterol. In transferosomal systems, cholesterol is often incorporated to modulate bilayer packing, reduce excessive membrane permeability, and improve vesicle stability, especially when formulations contain relatively high proportions of membrane-softening surfactants [11,19,26]. Its condensing effect on phospholipid bilayers can reduce local free volume and increase membrane cohesion, which may be beneficial for limiting premature drug leakage and improving storage stability [11,19]. At the same time, this stabilizing effect may come at the expense of membrane flexibility, since cholesterol can also increase bilayer order and reduce the extent of stress-induced deformation if included above an optimal range [11,26]. Thus, in transferosomes, cholesterol should not be regarded simply as a stabilizer, but as a bilayer-regulating excipient whose effect depends on its ratio relative to both phospholipids and edge activators. Recent experimental work illustrates this dual role: cholesterol-containing transferosomes may show improved morphological stability and altered release behavior, but not necessarily superior deformability or barrier transport [17,26].

Charge-inducing agents constitute another relevant auxiliary class because they affect vesicle–vesicle interactions, dispersion stability, drug association, and the interaction of transferosomes with biological substrates [12,19,27]. Positively charged additives such as stearylamine and negatively charged molecules such as dicetyl phosphate have been employed to modify the zeta potential of vesicular systems and thereby reduce aggregation through electrostatic repulsion [19,27]. In transferosomes, surface charge can also influence entrapment efficiency and skin interaction, particularly for ionizable drugs or when the formulation is intended to interact with negatively charged biological interfaces [19,27]. However, the effect of charge modifiers is not purely colloidal. Because these molecules become part of the interfacial environment of the vesicle, they may also alter membrane packing and permeability, sometimes enhancing retention and sometimes promoting instability depending on composition and charge density [19,27]. Studies with cationic transferosomes have shown that the inclusion of positively charged amphiphiles can substantially modify particle size, zeta potential, entrapment, stability, and permeation behavior, reinforcing the view that surface charge in transferosomes is a design variable rather than a passive descriptor [27].

The composition of the hydration medium is also more influential than is sometimes assumed. Parameters such as pH, ionic strength, buffer composition, and the presence of co-solutes can affect drug ionization, phospholipid hydration, vesicle size distribution, zeta potential, and the chemical stability of both the carrier and the payload [11,12]. This is particularly relevant for transferosomes because their membrane is already compositionally stressed by the presence of edge activators; consequently, changes in the aqueous environment may shift the balance between vesicle integrity and leakage more readily than in more rigid liposomal systems [11,12]. In drug-loaded formulations, the hydration medium may also influence drug localization between the aqueous core, bilayer, and interfacial domains, thereby affecting entrapment efficiency and release behavior [12]. For this reason, aqueous phase composition should be considered part of the formulation design rather than merely a procedural detail.

Auxiliary excipients used for stabilization during storage deserve special consideration, especially in view of the limited shelf stability commonly associated with deformable vesicular systems. Transferosomes, like other phospholipid dispersions, are prone to aggregation, fusion, hydrolysis, oxidation, and drug leakage during storage, and these issues may be exacerbated by the presence of surfactants that increase membrane mobility [11,12,28]. Freeze-drying has therefore emerged as an important strategy to improve long-term stability, but successful lyophilization requires the use of protective excipients capable of reducing membrane damage during freezing and dehydration [28,29,30]. Sugars such as sucrose and trehalose are commonly used as cryo- or lyoprotectants because they can preserve bilayer organization, reduce fusion upon drying, and improve redispersion after reconstitution [29,30]. Although much of the mechanistic understanding of these excipients comes from the broader liposome literature, the same principles are highly relevant to transferosomes, where structural preservation after drying is even more challenging due to the softer and more dynamic nature of the membrane [28,29,30]. Recent transferosome-specific work has confirmed that lyophilization can extend physicochemical stability without necessarily compromising release or permeation performance, provided that formulation and drying conditions are appropriately selected [28,31].

Taken together, these auxiliary components should not be viewed as marginal formulation details. In transferosomes, they often determine whether a composition that is theoretically attractive can actually function as a stable, reproducible, and pharmaceutically useful delivery system [11,12,19,26]. Cholesterol may improve bilayer integrity but dampen deformability; charge modifiers may enhance colloidal stability but alter interfacial behavior; hydration media may control ionization and membrane hydration; and cryo/lyoprotectants may be indispensable for preserving vesicle structure during storage [19,27,28,29,30]. Their influence is therefore highly contextual and must be interpreted in relation to the primary membrane composition, the drug load, and the intended route of administration. This makes auxiliary excipients an essential part of rational transferosome design rather than a secondary optimization layer. For clarity, the main formulation components of transferosomes and their functional roles are summarized in Table 1, with explicit listing of representative edge activators such as Tween 80, Span 80, sodium cholate, sodium deoxycholate, Tween 20, and Span 20.

### 2.4. Drug-Related Formulation Considerations

Drug-related variables are central to transferosome design because the physicochemical properties of the active ingredient strongly influence vesicle loading, intravesicular localization, membrane perturbation, release kinetics, and biological performance. Accordingly, transferosomes should not be treated as universally interchangeable carriers, but as formulation platforms whose behavior depends on the interplay between membrane composition and drug-specific attributes such as lipophilicity, ionization state, molecular size, solubility, and chemical stability [11,17,19]. This issue has become more evident in the recent literature, where transferosomes have been used not only for small transdermal actives but also for more challenging components, including hydrophilic drugs, peptides, and poorly soluble compounds intended for non-cutaneous barriers [17,21].

For hydrophilic drugs, transferosomal loading is generally associated with the aqueous core and interlamellar aqueous regions, although this simplified picture often fails to capture the importance of interfacial partitioning and hydration-dependent redistribution. In practice, encapsulation efficiency for water-soluble drugs is frequently constrained by the limited aqueous volume of nanosized vesicles, and this limitation becomes more pronounced when deformability is increased through surfactant-rich membranes [11,19]. This helps explain why hydrophilic payloads often require tighter control of vesicle size, lamellarity, phospholipid-to-edge-activator ratio, and external medium composition to balance deformability with retention. Recent transdermal studies using glucosamine-loaded transferosomes illustrate this point: the system showed successful nanoscale vesicle formation and therapeutic utility, but the formulation also required conversion into a gel to improve practical stability, underscoring that loading success alone does not guarantee pharmaceutical robustness [19,34].

Lipophilic drugs usually exhibit higher apparent entrapment in transferosomal systems because they preferentially partition into the hydrophobic region of the phospholipid bilayer. However, high bilayer affinity is not necessarily equivalent to optimal delivery behavior. Strong membrane association may slow drug release excessively, alter bilayer packing, compete with surfactants for interfacial space, and change vesicle mechanics in ways that affect both stability and barrier interaction [17,19]. Recent ocular work with curcumin-loaded transferosomes provides a good example of this formulation logic: the highly lipophilic drug achieved extremely high entrapment, minimal burst release, and enhanced tissue penetration, but these outcomes were clearly tied to the compatibility between the drug and the transferosomal membrane rather than to deformability alone [21]. Similar formulation-dependent effects have also been observed in topical and transdermal systems for lipophilic anti-inflammatory drugs, where cholesterol content, charge modifiers, and surfactant selection altered entrapment efficiency, stability, and permeation behavior [27].

Amphiphilic and weakly ionizable compounds often present the most formulation-sensitive behavior because they may partition among the aqueous core, bilayer interface, and external phase depending on pH, ionic strength, and membrane composition. In such cases, the measured entrapment efficiency may vary substantially depending on the analytical separation method, while apparently minor changes in formulation conditions can shift the equilibrium between vesicle-associated and freely dissolved drug [11,17,19]. This consideration is especially important in transferosomes because the surfactant-rich membrane environment can modify interfacial polarity and permeability more strongly than in conventional liposomes. Recent QbD-based work on dual-drug topical transferosomes for rheumatoid arthritis highlights this complexity: optimization of the formulation required simultaneous control of composition and process variables to achieve a useful balance among vesicle size, entrapment, deformability, and sustained release, illustrating that drug-related formulation behavior is often multidimensional rather than reducible to a single loading metric [22].

Drug molecular size is another major determinant of formulation feasibility. Transferosomes have long been promoted as carriers capable of improving the non-invasive delivery of macromolecules, especially peptides and proteins, but such applications impose substantially greater constraints than small-molecule delivery [17,35]. Larger biomolecules often suffer from low encapsulation efficiency, interfacial denaturation, limited stability during preparation, and more difficult permeation across intact barriers. Studies on insulin-loaded transferosomal gels illustrate both the promise and the challenge of this approach: factorial optimization showed that lipid ratio, surfactant ratio, and processing conditions strongly affected flux and entrapment, reinforcing the idea that macromolecular transferosome design must be highly formulation-specific [35]. More broadly, current transdermal reviews on peptide and protein delivery also make clear that vesicular encapsulation alone is rarely sufficient unless the carrier can preserve payload integrity while also providing a realistic permeation advantage across the barrier of interest [35,36].

The ionization state and chemical stability of the drug must also be considered from the outset. Drugs with pKa-dependent solubility or membrane affinity may behave very differently as formulation pH changes, and this can influence not only loading and retention but also release and tissue deposition. Likewise, transferosomes may protect some drugs from rapid degradation by partially isolating them from the external environment, yet they may also expose sensitive compounds to new stresses associated with lipid oxidation, surfactant-rich interfaces, and storage-related membrane rearrangements [11,17,19]. For this reason, formulation screening should not be driven solely by initial entrapment efficiency or short-term permeation results; drug stability over preparation, storage, and application conditions is equally important for judging whether a transferosomal system is pharmaceutically meaningful [17,19].

Overall, drug-related formulation considerations are not secondary optimization details but one of the main determinants of transferosome performance. Hydrophilic, lipophilic, amphiphilic, and macromolecular payloads interact with transferosomal membranes in fundamentally different ways, and these differences directly affect entrapment, retention, release, deformability, and barrier transport [11,17,19,21,22,27,34,35,36]. Transferosomes should therefore be developed as drug-specific systems rather than as generic ultradeformable vesicles, and meaningful interpretation of their performance requires that formulation outcomes always be read in light of the physicochemical behavior of the incorporated drug.

### 2.5. Main Preparation Methods and Process-Related Variables

Once the composition of a transferosomal system has been selected, the preparation method becomes a second major determinant of its final properties. This is particularly important because transferosomes are not equilibrium structures defined only by their ingredients; rather, they are process-sensitive vesicular systems whose size distribution, lamellarity, entrapment efficiency, surface characteristics, and deformability are strongly influenced by the way the bilayer is formed, hydrated, downsized, and homogenized [12,17,19,37]. Accordingly, formulation design in transferosomes must be understood as the combination of compositional design and process engineering, rather than as a purely composition-driven exercise [12,19].

The most widely used preparation route remains thin-film hydration, also referred to as lipid film hydration or rotary evaporation. In this method, phospholipids, edge activators, and lipophilic drug components are dissolved in an organic solvent or solvent mixture, followed by solvent evaporation to form a thin lipid film that is subsequently hydrated with an aqueous phase [17,19,37,38]. This approach is popular because of its simplicity, broad compatibility with diverse lipid compositions, and ability to generate vesicles suitable for subsequent size reduction and optimization. However, thin-film hydration is also highly sensitive to process conditions such as solvent composition, residual solvent removal, hydration temperature, hydration time, and agitation regime, all of which can influence vesicle formation and reproducibility [12,17,19]. In recent transferosome studies, thin-film hydration has remained the dominant method, not because it is universally ideal, but because it offers a flexible experimental platform for screening phospholipid–surfactant combinations and for coupling with downstream optimization steps such as sonication or extrusion [19,37,38].

Another frequently used strategy is ethanol injection or related solvent-displacement methods, in which the organic phase containing lipids is injected into an aqueous medium under controlled mixing conditions. Compared with film hydration, ethanol injection may offer faster vesicle formation and can reduce some of the handling complexity associated with film deposition and rehydration, although the final properties remain strongly dependent on injection rate, solvent fraction, temperature, mixing intensity, and post-processing [12,17]. More broadly, the growing interest in process simplification and scalability has increased attention on preparation routes that are more amenable to controlled and continuous manufacturing, even though thin-film hydration continues to dominate the experimental literature [12,19].

Regardless of the initial vesicle-forming method, post-formation size reduction is usually required to obtain nanoscale dispersions with acceptable uniformity. In transferosome preparation, this is commonly achieved through sonication, extrusion through polycarbonate membranes, or homogenization-based approaches [12,17,19]. Sonication is often used because it is convenient and effective for reducing vesicle size, but excessive sonication may promote drug leakage, induce local overheating, or alter membrane organization, especially in formulations with highly fluid bilayers [12,17,19]. Extrusion can provide narrower size distributions and better control over vesicle diameter, but its outcome depends on membrane pore size, number of extrusion cycles, temperature, and the mechanical resistance of the vesicle population being processed [12,19]. Because transferosomes are deliberately designed to be deformable, the choice of downsizing method is particularly consequential: the same formulation may exhibit different apparent size, distribution, and even deformability depending on whether it was probe-sonicated, bath-sonicated, or extruded under controlled pressure [12,17,19].

The importance of process variables is increasingly evident in recent optimization studies. In a Box–Behnken design study on silymarin-loaded transferosomes, phospholipid concentration, surfactant concentration, and sonication time were found to significantly affect encapsulation efficiency and drug release, illustrating how process-related and compositional variables interact rather than act independently [20]. Similarly, recent design-based optimization of nano-transferosomes co-loaded with pioglitazone and eprosartan mesylate identified phospholipid amount, surfactant level, solvent ratio, and sonication time as key determinants of entrapment efficiency and transdermal flux, demonstrating that process variables can influence therapeutic output as directly as formulation ingredients [39]. In another example, DOE-assisted optimization of tioconazole-loaded transferosomal hydrogel highlighted the importance of phospholipid and sodium deoxycholate levels in controlling particle size, polydispersity, and entrapment efficiency, reinforcing the idea that process optimization in transferosome development is best approached as a multivariable problem [40].

Processing conditions also affect drug-specific outcomes. In lidocaine-loaded transferosomal gels, film hydration followed by formulation optimization showed that vesicle properties and subsequent skin permeation were strongly influenced not only by the lipid/surfactant system but also by how the vesicles were incorporated into the final semisolid dosage form [41]. Likewise, in hydrocortisone transferosomes, optimization of the film hydration process and the phospholipid-to-surfactant balance markedly altered encapsulation efficiency, drug release, elasticity, and ex vivo permeation [42]. These examples illustrate an important principle: in transferosomal systems, process variables do not simply refine a finished composition; they actively shape the structural state in which that composition is expressed.

From a translational standpoint, the preparation method also matters because not all laboratory-scale procedures are equally compatible with scale-up, reproducibility, and industrial manufacturing. Thin-film hydration is useful for early formulation screening, but it is less attractive for large-scale continuous production because of solvent-handling complexity, batch dependence, and scale-related variability [12,19]. This is one reason why recent reviews increasingly discuss high-pressure homogenization, microfluidic adaptation, and other scalable lipid-processing strategies as potential future directions for transferosome production, even if they are not yet dominant in the current literature [12,37]. The broader implication is that a preparation method should not be judged only by whether it produces small vesicles or high entrapment under laboratory conditions, but also by whether it can support robust batch-to-batch quality and realistic pharmaceutical development.

Overall, the preparation of transferosomes is best understood as a critical design stage in which vesicle properties are generated rather than merely revealed. The initial method of vesicle formation, the choice of downsizing strategy, and the precise control of process variables such as temperature, solvent system, hydration conditions, mixing intensity, and sonication or extrusion parameters all contribute to the final physicochemical and functional profile of the formulation. For this reason, process-related variables should be considered integral critical formulation determinants, not procedural details, in any rigorous evaluation of transferosomal systems. The most common preparation methods used for transferosomes and their main effects on vesicle properties are summarized in Table 2.

## 3. Critical Quality Attributes and Characterization

A meaningful evaluation of transferosomes requires more than reporting their composition and preparation method. Because these vesicular systems are highly sensitive to both formulation and process variables, their performance must be interpreted through critical quality attributes (CQAs) that reflect vesicle structure, colloidal behavior, drug incorporation, membrane mechanics, and storage stability. In this context, characterization is not a descriptive checklist, but the basis for establishing whether a given formulation is reproducible, functionally relevant, and suitable for comparison with other deformable vesicular systems. This section discusses the main CQAs of transferosomes and the analytical approaches used to assess them, with emphasis on parameters directly linked to formulation quality and delivery performance.

### 3.1. Vesicle Size, Size Distribution, and Surface Charge

Vesicle size, size distribution, and surface charge are among the most informative and most frequently reported quality attributes in transferosomal systems because they directly affect colloidal stability, drug loading behavior, membrane deformability, barrier interaction, and storage performance [17,37,43]. These parameters are not merely descriptive outputs of formulation; rather, they are structural indicators of how composition and process variables are translated into a vesicular system with a defined physicochemical identity [17,37]. Recent reviews continue to regard them as part of the minimum characterization package required for meaningful comparison between transferosomal formulations, particularly in dermal, transdermal, and ocular delivery studies [17,37].

Vesicle size is especially important because it influences both the internal architecture of the carrier and its interaction with biological barriers [17,43]. In transferosomes, particle size affects surface area, internal aqueous volume, curvature stress, interfacial contact with tissues, and the likelihood of aggregation during storage [17,43]. Smaller vesicles are often associated with improved contact with biological surfaces and, in many topical applications, with more favorable penetration and deposition behavior; however, very small vesicles may also display greater curvature-driven instability or altered drug retention, particularly when the bilayer is highly fluid [17,43]. Vesicle size should therefore not be interpreted in isolation as a marker of formulation superiority, but as one element of a broader structure–property relationship [17,37,43]. General nanocarrier literature likewise identifies size as a major determinant of delivery behavior, while recent transferosome studies continue to optimize size as a key response variable rather than treating it as a passive measurement [43].

Equally important is size distribution, which is commonly expressed through the polydispersity index (PDI). In transferosomal systems, PDI serves as a practical indicator of population uniformity and therefore of formulation reproducibility, colloidal robustness, and the representativeness of the measured mean size [17,37,44]. A low-to-moderate PDI is generally preferred because broad or multimodal size distributions complicate the interpretation of deformability, drug release, permeation, and stability studies [17,44]. This issue is particularly relevant for transferosomes because soft vesicular systems are sensitive to post-processing conditions, surfactant content, and storage-induced changes, all of which may broaden size distribution even when the average diameter appears acceptable [17,37]. Recent primary studies continue to use PDI as a critical optimization endpoint alongside vesicle size and entrapment efficiency, confirming that narrow size distribution is a central quality attribute rather than a merely cosmetic formulation goal [32,45,46].

From an analytical standpoint, vesicle size and PDI are most commonly determined by dynamic light scattering (DLS), which remains the standard technique in the transferosome literature because it is rapid, widely accessible, and suitable for dispersed nanosystems [17,44,47,48]. However, DLS reports an intensity-weighted hydrodynamic diameter and is highly sensitive to the presence of even a small number of larger particles or aggregates [47,48]. For this reason, apparent changes in size or PDI may reflect dilution conditions, multiple scattering, dust contamination, aggregation, or sample heterogeneity rather than genuine formulation differences [47,48]. Recent methodological analyses continue to emphasize that DLS is indispensable but easily overinterpreted when used as the sole measure of particle population [44,47,48]. This caution is particularly relevant for transferosomes, whose softness and possible multimodal distributions can complicate the relationship between measured hydrodynamic size and actual vesicle morphology [17,44]. Accordingly, DLS data should ideally be interpreted together with complementary structural techniques such as electron microscopy or cryogenic imaging when more precise conclusions about vesicle architecture are needed [17,44,48].

Surface charge, usually reported as zeta potential, is another key quality attribute because it provides indirect information on electrostatic stabilization, interfacial composition, and possible interactions with the surrounding medium [43,48]. In transferosomes, zeta potential is influenced by phospholipid headgroups, charge-inducing agents, bile salts or ionic surfactants, buffer composition, pH, ionic strength, and adsorbed drug or biomolecules [17,37,48]. More negative or more positive zeta-potential values are often interpreted as indicators of improved dispersion stability, but zeta potential should not be used as a universal surrogate for shelf stability [48]. In soft vesicular systems, steric stabilization from non-ionic surfactants, hydration forces, and membrane fluidity may contribute substantially to stability even when the absolute zeta potential is only moderate [17,48]. Both the general nanoparticle characterization literature and recent transferosome-focused studies support the view that zeta potential is a useful but context-dependent parameter whose meaning depends strongly on the medium conditions and the formulation architecture [48,49].

In practical formulation development, zeta potential is also valuable because it can reveal how transferosomes respond to physiologically relevant environments. A recent ocular study showed that transferosomes suspended in simulated healthy and dry-eye tear fluids underwent marked changes in vesicle size, PDI, and zeta potential relative to measurements made in water [49]. This finding is relevant beyond ophthalmic delivery, since dermal and mucosal formulations may also behave differently in saline, buffer, biological fluid, or gel matrices than in purified water [17,49]. Surface charge should therefore be interpreted not only as an intrinsic formulation property, but also as an environment-sensitive descriptor of colloidal behavior [48,49].

Recent transferosome studies further illustrate how size, PDI, and zeta potential function together as critical optimization endpoints. For example, cefepime-loaded nano-transferosomes optimized by statistical design were reported with a vesicle size of approximately 223 nm, a PDI of 0.163, and a zeta potential of about −20.8 mV, values interpreted as consistent with a physically stable nanosystem suitable for incorporation into a transdermal chitosan gel [45]. In other recent examples, optimized transferosomal systems developed for melanoma-oriented topical delivery and antifungal gel formulations were likewise characterized through this same core triad of metrics, reinforcing that vesicle size, size distribution, and surface charge remain central decision variables in current transferosome development rather than legacy descriptors inherited from older liposome literature [32,46].

Taken together, vesicle size, size distribution, and surface charge should be regarded as foundational CQAs in transferosome research [17,37]. They are essential not only because they help describe the formulation, but because they determine whether subsequent results on entrapment, deformability, release, permeation, and stability can be interpreted with confidence [17,37,43]. For this reason, these parameters should be measured under clearly reported conditions, discussed in relation to both formulation composition and dispersion medium, and, whenever possible, supported by orthogonal characterization methods [44,47,48]. A transferosome formulation cannot be considered adequately characterized if its apparent performance is discussed without a robust understanding of these three interrelated attributes [17,37].

### 3.2. Encapsulation Efficiency and Drug Loading

Encapsulation efficiency (EE) and drug loading are core quality attributes in transferosomal systems because they determine how much of the administered drug is actually associated with the vesicles and how efficiently the carrier mass is being used for therapeutic transport [17,19,47,50]. Although the two terms are sometimes used interchangeably in the transferosome literature, they describe different formulation properties. Encapsulation efficiency refers to the fraction or percentage of the initial drug input that becomes vesicle-associated after preparation, whereas drug loading reflects the amount of incorporated drug relative to the lipid or total carrier mass [17,50]. This distinction is important because a formulation may show high EE simply by starting from a low drug dose, yet still have modest practical loading capacity. Conversely, increasing drug input may raise loading while reducing EE if the vesicle system approaches saturation or becomes unstable [47,50].

In transferosomes, EE and loading are strongly governed by the physicochemical compatibility between the drug and the vesicular architecture. Hydrophilic compounds tend to partition preferentially into the aqueous core or interlamellar aqueous regions, whereas lipophilic drugs generally associate with the hydrophobic bilayer. Amphiphilic compounds may distribute across both interfacial and internal regions depending on ionization state, membrane composition, and medium conditions [17,19]. Because transferosomes incorporate edge activators that soften the membrane and alter interfacial packing, the drug-associated fraction may be particularly sensitive to surfactant type, phospholipid-to-surfactant ratio, cholesterol content, and hydration medium composition [17,19,47]. As a result, EE and loading should be interpreted as emergent properties of the full formulation system rather than as inherent features of the drug alone.

Methodologically, accurate determination of EE requires a reliable separation of vesicle-associated drug from free or non-encapsulated drug before quantification. A recent systematic review of liposomal EE determination emphasized that the most common analytical approaches include ultracentrifugation, ultrafiltration, size-exclusion chromatography, equilibrium dialysis, and reverse dialysis, each with distinct biases and suitability depending on vesicle size, drug properties, membrane softness, and formulation viscosity [50]. That review also stressed that accurate EE determination generally requires measurement of at least two of the following three quantities: total drug, free drug, or encapsulated drug [50]. This methodological point is highly relevant to transferosomes, since their deformability and surfactant-rich membranes may complicate separation procedures more than in rigid liposomal systems, increasing the risk of drug leakage, vesicle disruption, or incomplete recovery during analysis [47,50].

In practice, many transferosome studies still rely on centrifugation- or ultracentrifugation-based indirect methods, in which the unentrapped drug is quantified in the supernatant and EE is calculated by difference [19,21,45,51,52]. These approaches are convenient and widely used, but they are not analytically neutral. Centrifugation conditions that are too harsh may deform or disrupt vesicles, whereas insufficient centrifugal force may fail to separate small or highly deformable carriers efficiently [47,50]. For this reason, the measured EE may partly reflect the analytical method itself rather than the true formulation state. This is one of the main reasons why recent methodological literature recommends more careful validation of separation protocols, especially when dealing with soft lipid nanocarriers [47,50].

Recent primary studies illustrate how widely EE can vary across transferosomal systems depending on both the drug and the formulation design. In curcumin-loaded transferosomes for ocular delivery, Barbalho et al. reported an EE greater than 99.96%, which is consistent with the strong bilayer affinity of this hydrophobic compound and the compatibility between curcumin and the transferosomal membrane environment [21]. In contrast, papaverine hydrochloride transferosomes prepared for the topical treatment of erectile dysfunction showed EE values ranging from about 18% to nearly 72%, with the optimized system reaching approximately 72% [52]. Likewise, cefepime nano-transferosomes incorporated into chitosan gel were optimized with EE as a key response variable, while ocular tonabersat transferosomes and melanoma-oriented genistein transferosomes were also characterized in part by their ability to achieve pharmaceutically meaningful encapsulation [45,46,51]. These examples show that EE values are formulation-specific and should not be compared across studies without considering drug polarity, membrane composition, preparation method, and separation technique.

Drug loading is often less extensively discussed than EE in the transferosome literature, but it is equally important from a pharmaceutical standpoint because it determines how much active compound can be delivered per unit mass of carrier [17,50]. This is especially relevant for systems intended for topical, ocular, or transdermal administration, where dosage volume may be limited and excessive excipient burden may reduce tolerability or complicate translation. In the curcumin ocular study, for example, increasing the drug loading percentage increased both loading and entrapment up to the investigated range, illustrating that the formulation had not yet exceeded the membrane’s practical loading capacity [21]. However, such behavior cannot be generalized: in many vesicular systems, loading beyond an optimal threshold may alter membrane packing, promote drug crystallization or leakage, broaden size distribution, and reduce physical stability [47,50]. Therefore, drug loading should always be interpreted together with vesicle size, PDI, zeta potential, and stability data rather than as a standalone metric.

Another important consideration is that high EE is not necessarily predictive of superior delivery performance. A formulation may encapsulate a large fraction of the drug but release it too slowly, retain it too strongly within the bilayer, or lose it during storage or biological exposure. Conversely, a moderate EE may still be pharmaceutically useful if the formulation shows adequate stability, appropriate release kinetics, and meaningful tissue delivery [17,32,50]. This is evident in recent transferosome-based gel systems such as optimized fluconazole and silymarin nano-transferosomal formulations, where EE was one optimization target among several and had to be interpreted alongside release behavior, permeation, and therapeutic output [20,32]. Thus, EE and drug loading should be treated as necessary but not sufficient indicators of formulation quality.

From a quality perspective, meaningful reporting of EE and drug loading in transferosomes requires a transparent description of the analytical workflow. At a minimum, studies should specify whether EE was determined directly or indirectly, how the free drug was separated from vesicle-associated drug, what drug assay was used, whether vesicle disruption was validated before total drug quantification, and under what medium conditions the analysis was conducted [47,50]. Without such information, it becomes difficult to compare formulations or to determine whether differences in reported EE reflect genuine formulation improvements or simply methodological variation. This issue has been clearly highlighted in recent analytical reviews and remains one of the main barriers to robust comparison across lipid nanocarrier studies [47,50].

Overall, EE and drug loading are indispensable CQAs in transferosome characterization because they connect formulation design with therapeutic practicality. However, their value depends on rigorous analytical determination, careful interpretation in light of drug–membrane interactions, and integration with other physicochemical and performance parameters [17,19,47,50]. In transferosomal systems, these attributes should therefore be viewed not as isolated percentages, but as part of a broader framework linking composition, process, vesicle structure, release behavior, and translational relevance.

### 3.3. Morphology and Internal Organization

Morphology and internal organization are critical yet frequently underinterpreted attributes in transferosomal systems because they provide direct information on how formulation composition and processing are translated into a vesicular architecture. Unlike average size or zeta potential, which are indirect descriptors of colloidal behavior, morphological analysis can reveal whether the dispersion is truly vesicular, whether the vesicles are predominantly spherical or irregular, whether they are uni- or multilamellar, and whether drug incorporation or processing has induced structural heterogeneity [17,47,53,54]. In transferosomes, these issues are particularly important because the membrane is intentionally softened by edge activators, making the final nanostructure more sensitive to preparation conditions, drug loading, osmotic stress, and storage history than in more rigid liposomal systems [17,47].

In most studies, transferosomes are described as approximately spherical or quasi-spherical vesicles, often with unilamellar or oligolamellar organization, but this simplified description should be treated with caution. Morphology in soft lipid nanocarriers is highly dependent on the characterization technique used and on sample preparation artifacts [47,54]. Negative-stain transmission electron microscopy (TEM) remains one of the most frequently used methods in the transferosome literature because it is accessible and useful for confirming vesicle formation and approximate shape. However, staining, dehydration, and vacuum exposure may flatten, collapse, or distort soft vesicles, meaning that TEM images do not always represent the native hydrated morphology of the system [47,54]. This limitation is well recognized in broader lipid-nanoparticle characterization literature and is directly relevant to transferosomes, where membrane deformability makes structural artifacts more likely during conventional electron microscopy workflows [47,53].

For this reason, cryo-transmission electron microscopy (cryo-TEM) is generally considered the most informative imaging technique for evaluating the native morphology of transferosomes and related soft vesicular systems. By vitrifying the sample in its hydrated state and avoiding staining-induced collapse, cryo-TEM allows direct visualization of vesicle contour, lamellarity, membrane continuity, and, in some cases, internal structural heterogeneity [21,47,53]. Recent reviews on lipid nanoparticle structure emphasize that cryo-TEM is especially powerful when combined with scattering techniques because it can show the real morphology, while SAXS or SANS provides complementary information on internal organization and average nanostructural features across the population [4]. This combined structural approach is highly relevant for transferosomes, whose functional behavior depends not only on mean size but also on whether the vesicles retain a coherent bilayer organization under the conditions used for delivery [17,53].

Internal organization in transferosomes mainly refers to bilayer arrangement, lamellarity, membrane thickness, and the spatial distribution of drug-associated domains within the vesicle. These parameters are rarely captured adequately by DLS alone and often require orthogonal techniques. Cryo-TEM can reveal whether the vesicles are predominantly unilamellar, multilamellar, or structurally heterogeneous, while small-angle X-ray scattering (SAXS) can provide population-averaged information on bilayer spacing, internal periodicity, and structural transitions that are not obvious from imaging alone [47,53]. More general structural reviews of lipid nanoparticles have emphasized that no single technique is sufficient to resolve morphology and internal organization comprehensively; rather, imaging and scattering methods should be interpreted together to avoid overreliance on a few representative micrographs [53]. This point is especially important in transferosome research, where a formulation may appear morphologically acceptable in TEM images while still exhibiting hidden internal heterogeneity or composition-dependent nanostructural rearrangements [17,53].

Recent transferosome studies illustrate the value of morphological analysis beyond simple visual confirmation. In curcumin-loaded ocular transferosomes, Barbalho et al. used cryo-TEM to support the presence of vesicular nanostructures and linked morphology to penetration behavior in different ocular tissues, highlighting that structural assessment can be functionally informative rather than merely illustrative [21]. Likewise, in a later ocular formulation study, Bhujbal et al. characterized tonabersat-loaded transferosomes and used imaging as part of a broader physicochemical analysis to interpret how the system behaved under application-relevant conditions [51]. These studies are important because they move morphology from a decorative characterization step toward a mechanistically useful one, particularly in biological environments where vesicle integrity may change after contact with tear fluid or tissue interfaces [21,51].

Morphological analysis is also valuable when transferosomes are combined with secondary payloads or hybrid structures. In microneedle-assisted transferosome formulations containing gold nanoparticles, TEM was used not only to confirm vesicle formation but also to visualize the successful incorporation of the model payload and the preservation of vesicular morphology after loading [55]. This type of evidence is particularly useful because internal organization may change when the drug or added nanocomponent partitions strongly into the bilayer or disrupts membrane curvature. Accordingly, morphology should be viewed as a sensitive indicator of formulation compatibility and not only as confirmation that nanosized particles exist [17,55].

Another complementary technique is atomic force microscopy (AFM), which can provide information on surface topography, vesicle shape after deposition, and qualitative mechanical behavior. AFM has been increasingly used in soft nanocarrier characterization because it can visualize nanoscale surface features and, depending on the mode employed, offer indirect insight into deformability and mechanical response [47,56]. However, AFM images must also be interpreted carefully in transferosomes because adsorption onto a solid substrate may induce flattening or deformation, particularly in highly flexible vesicles. Thus, AFM is best regarded as a complementary technique rather than a standalone method for defining the native morphology of transferosomes [47,56].

In summary, morphology and internal organization should be considered core structural quality attributes in transferosome characterization. They are essential for confirming the vesicular nature of the system, identifying lamellarity and heterogeneity, interpreting the impact of drug loading or auxiliary excipients, and understanding whether the formulation retains a coherent nanostructure under relevant conditions [17,21,47,51,53,54,55]. For this reason, a rigorous characterization strategy should go beyond reporting a few negative-stain TEM images and should, whenever possible, combine imaging with orthogonal structural methods such as cryo-TEM and SAXS. In transferosome research, morphology is not a cosmetic descriptor; it is one of the clearest windows into whether the formulation truly possesses the nanostructural organization required to support its claimed delivery function [47,53].

### 3.4. Physical and Chemical Stability

Physical and chemical stability are decisive quality attributes in transferosomal systems because they determine whether the vesicular structure, drug content, and functional performance are preserved throughout storage, handling, and application [17,47,57,58]. This issue is especially important in transferosomes, whose bilayers are intentionally softened by edge activators to promote deformability. While this membrane adaptability is central to their delivery function, it also makes them intrinsically more vulnerable to aggregation, fusion, drug leakage, hydrolysis, oxidation, and other storage-related changes than more rigid vesicular systems [17,47,58]. For this reason, stability in transferosomes should not be treated as a secondary formulation check, but as a central criterion for judging whether a promising laboratory formulation has realistic pharmaceutical value [17,47].

From a physical stability standpoint, the main concerns in transferosomal dispersions include changes in vesicle size, polydispersity, zeta potential, lamellarity, sedimentation behavior, fusion, and leakage of the encapsulated drug [17,47,58]. These alterations may arise from colloidal instability, membrane reorganization, osmotic imbalance, surfactant redistribution, or mechanical stress during storage and transport [47,58]. In practice, size increase and broadening of size distribution are often early indicators of destabilization, particularly in formulations stored as aqueous dispersions. Because transferosomes are soft and highly dynamic systems, even modest shifts in these parameters may indicate underlying changes in vesicle organization that later affect drug release or permeation behavior [47,58]. Recent general reviews on liposomal formulations likewise emphasize that long-term vesicle integrity depends strongly on lipid composition, surfactant effects, hydration medium, temperature, and storage conditions [57].

Chemical stability in transferosomes is governed primarily by the susceptibility of phospholipids and incorporated compounds to degradation reactions. The two most important degradation pathways for phospholipid-based carriers are hydrolysis and oxidation [57,59]. Hydrolysis may cleave ester bonds in phospholipids, generating lysolipids and free fatty acids that alter bilayer packing and promote leakage or structural collapse. Oxidation, especially in unsaturated phospholipids, may generate peroxides and secondary degradation products that compromise membrane integrity, affect zeta potential, destabilize drug association, and reduce product safety or efficacy [57,59]. These risks are particularly relevant in transferosomes because phospholipid bilayers softened by edge activators may be more sensitive to environmental stressors such as light, oxygen, heat, and moisture than more condensed lipid systems [17,57,59]. A recent review of oxidative stability in lipid formulations underscores that oxidation is not merely a lipid chemistry concern but a major determinant of formulation shelf life, with implications for excipient selection, antioxidant strategy, and packaging design [59].

The drug itself may also contribute to instability. Some payloads are prone to crystallization, hydrolysis, oxidation, isomerization, or partitioning changes during storage, while others may destabilize the bilayer by altering membrane packing or competing with surfactants at the interface [17,47]. As a result, acceptable short-term physicochemical characterization immediately after preparation does not guarantee that the formulation will remain suitable over time. This is why stability evaluation in transferosomes should ideally include not only vesicle-related parameters such as size, PDI, and zeta potential, but also drug-related parameters such as drug content, entrapment retention, pH, and, when relevant, chemical integrity of the active compound [17,47,57]. Recent transferosome studies increasingly include these broader stability panels, especially when the formulation is intended for topical or transdermal use over prolonged storage periods [29,31,46].

Temperature is one of the most influential storage variables. Refrigerated conditions are often preferred because they slow down hydrolysis, oxidation, vesicle fusion, and drug leakage; however, low temperatures may also promote phase transitions or destabilization in specific lipid compositions [47,57]. Storage at room temperature may be more practical from a translational standpoint, but it can accelerate chemical degradation and colloidal change, particularly in dispersions containing unsaturated phospholipids or oxidation-sensitive actives [57,59]. This is why many studies evaluate transferosome stability under at least two temperature conditions, commonly around 4–8 °C and 25 °C, and compare changes in size, zeta potential, PDI, pH, and drug content over time [29,46]. In genistein-loaded transferosomal hydrogel, for example, the optimized formulation showed strong physical and chemical stability over 12 months at both 5 °C and 25 °C, with monitored changes in particle size, PDI, zeta potential, pH, and drug content used to support formulation robustness [46].

Because aqueous dispersions remain vulnerable to long-term instability, drying strategies, especially lyophilization, have become increasingly important in transferosome development [29,47,57]. Freeze-drying can significantly improve storage stability by reducing hydrolytic degradation and limiting vesicle fusion in the absence of bulk water, but it also introduces its own challenges. Freezing and dehydration can damage bilayer structure, induce fusion, change vesicle size upon reconstitution, and cause drug leakage unless suitable cryo- or lyoprotectants are included [29]. This issue is well established in the broader liposome literature, where lyophilization remains both necessary and technically challenging for many formulations [29]. The same principle applies, often more critically, to transferosomes because of their softer membranes. A recent study on Panax notoginseng total saponins-loaded transferosomes showed that lyophilization improved physicochemical stability without adversely affecting ex vivo or in vivo skin permeation, supporting the practical value of this approach when the formulation and reconstitution conditions are properly optimized [31].

Semisolid incorporation may also contribute to physical stability. When transferosomes are embedded in gels or hydrogel matrices, the continuous phase may reduce vesicle mobility, limit aggregation, improve residence time, and protect the formulation against sedimentation-related instability [17,47]. For example, transferosome-stabilized hydrogels have been reported to maintain acceptable pH and sedimentation behavior under freeze–thaw conditions, suggesting that matrix incorporation can improve formulation robustness beyond the dispersed vesicle state alone [60]. Similarly, transferosomal chitosan gels and Carbopol-based systems have been shown to preserve physicochemical properties over storage while also offering more practical dosage forms for topical application [45,46]. These observations are relevant because many transferosome formulations are unlikely to reach pharmaceutical use as simple aqueous dispersions and instead will require a secondary dosage form with its own impact on stability [45,46,60].

A rigorous stability evaluation should therefore distinguish between physical stability, chemical stability, and functional stability. Physical stability concerns vesicle integrity and colloidal behavior; chemical stability concerns preservation of lipid and drug composition; and functional stability concerns whether the formulation still retains meaningful release, permeation, and biological performance after storage [17,29,47,57]. This distinction is important because a transferosomal system may remain visually acceptable while losing drug content, undergo minimal size change while suffering membrane oxidation, or retain entrapment while showing altered permeation after storage. Thus, stability studies should be designed to capture not only conventional physicochemical parameters but also the aspects of performance most relevant to the intended route of administration and dosage form [17,31,46,57].

Physical and chemical stability are not merely supportive characterization endpoints in transferosome research; they are essential determinants of translational feasibility. A formulation that achieves excellent size, entrapment efficiency, and deformability immediately after preparation may still be pharmaceutically unsuitable if it cannot maintain vesicle integrity, drug content, and functional behavior over time [17,47,57]. For this reason, stability assessment should be integrated early into transferosome design and interpreted as a core part of quality evaluation, not as a final confirmatory step.

### 3.5. Analytical Methods Used in Transferosome Characterization

A meaningful characterization of transferosomes requires an orthogonal analytical strategy, because no single technique can adequately describe all the structural, colloidal, interfacial, and functional attributes of these vesicular systems [17,47,48,53]. This point is especially important for transferosomes, whose membranes are intentionally softened by edge activators and are therefore more susceptible to preparation artifacts, environmental changes, and method-dependent interpretation than conventional rigid vesicles [17,47]. Accordingly, analytical characterization should be viewed not as a checklist of isolated measurements, but as an integrated framework linking vesicle size, population homogeneity, surface charge, internal organization, drug association, thermal behavior, and performance-related properties [17,47,48,53].

At the most basic level, dynamic light scattering (DLS) and electrophoretic light scattering remain the most widely used techniques for routine transferosome characterization because they provide rapid information on hydrodynamic size, polydispersity index (PDI), and zeta potential [17,47,48]. These methods are practical and widely accessible, which explains their central role in both review literature and recent primary studies on transferosomes [17,19,47,61]. However, DLS provides an intensity-weighted hydrodynamic diameter and is highly sensitive to dust, aggregates, and multimodal populations, while zeta-potential measurements depend strongly on pH, ionic strength, conductivity, and sample dilution [47,48]. For this reason, DLS and zeta potential are indispensable screening tools, but they should not be treated as standalone proof of nanostructural quality [47,48]. Their main strength lies in rapid comparative analysis, whereas their main limitation lies in the risk of overinterpreting simplified averages in structurally complex or highly deformable dispersions [47,48].

To confirm vesicle formation and assess morphology, the transferosome literature relies heavily on electron microscopy, particularly transmission electron microscopy (TEM) and, where available, cryo-TEM [17,53,61]. Conventional TEM is useful for visual confirmation of vesicle presence, approximate shape, and coarse structural homogeneity, but negative staining, drying, and vacuum exposure may distort soft vesicles and alter their apparent morphology [47,53]. By contrast, cryo-TEM preserves the hydrated state more faithfully and is therefore more informative for evaluating native morphology, membrane continuity, and lamellarity [53]. In current transferosome practice, TEM is still more commonly reported than cryo-TEM because of accessibility, but the latter is methodologically superior for soft lipid nanocarriers when native-state structural interpretation is important [47,53]. Recent transferosome studies, such as phloretin and curcumin formulations, have continued to use TEM or cryo-TEM as part of a broader characterization package rather than as isolated visual evidence [21,61].

Complementary information on vesicle topography and deposited-shape behavior may be obtained by atomic force microscopy (AFM) [47,56]. AFM is useful because it can visualize nanoscale surface features and provide qualitative information about vesicle deformation after surface adsorption. However, as with TEM, AFM is susceptible to preparation-induced artifacts, especially when highly flexible vesicles flatten on the substrate [47,56]. For this reason, AFM is best interpreted as a complementary surface-sensitive technique rather than definitive proof of native morphology. In practice, its value is greatest when used alongside DLS and electron microscopy to compare relative structural behavior across formulations rather than to define vesicle architecture on its own [47,56].

Beyond imaging, several thermal and spectroscopic methods are commonly used to probe drug–excipient interactions and the physical state of the incorporated drug. Differential scanning calorimetry (DSC) is frequently employed to detect shifts in transition temperatures, disappearance of drug-melting endotherms, or changes in bilayer organization, thereby providing indirect evidence of drug incorporation or altered membrane packing [17,21,61]. Fourier-transform infrared spectroscopy (FTIR) is widely used to investigate possible interactions between the drug and excipients through band shifts or intensity changes, while X-ray diffraction (XRD) can help determine whether the drug remains crystalline or becomes amorphous/disordered after incorporation into the vesicular system [21,61]. These techniques are particularly useful in transferosome studies because they provide information that cannot be inferred from size or zeta potential alone. Recent formulations, such as phloretin-loaded transferosomes, explicitly combined TEM, DSC, FTIR, and XRD to support conclusions about vesicle structure, compatibility, and drug state within the system [61].

Where a deeper understanding of internal organization is needed, small-angle scattering methods such as SAXS and, less commonly, SANS, can provide population-averaged information on bilayer thickness, internal spacing, nanostructural order, and lamellarity that is not accessible from a few microscopy images alone [53]. Reviews of lipid nanoparticle structure have emphasized that imaging and scattering methods should ideally be interpreted together, because microscopy offers direct visualization of selected particles, whereas scattering provides statistically broader information about the whole dispersion [53]. This combined strategy is particularly relevant to transferosomes, whose internal organization may be subtly affected by drug incorporation, surfactant ratio, cholesterol content, or storage-induced rearrangement without necessarily producing obvious changes in mean size [17,53].

Determination of encapsulation efficiency (EE) and drug loading requires a different analytical layer, centered on separation and quantification. Recent methodological analysis of liposomal EE determination has highlighted that accurate quantification depends not only on the drug assay itself, often based on HPLC or UV–Vis spectroscopy, but also on how free and vesicle-associated drug are separated before analysis [50]. Techniques such as ultracentrifugation, ultrafiltration, size-exclusion chromatography, and dialysis can all be used, but each introduces possible biases related to vesicle disruption, incomplete separation, or drug adsorption [50]. In transferosomes, these issues are even more relevant because deformable vesicles may be more sensitive to mechanical or osmotic stress during the separation step [17,47,50]. Therefore, drug quantification in transferosomal systems should always be interpreted together with the chosen separation method rather than as a purely analytical endpoint [50].

Analytical characterization also extends into function-oriented methods, especially when the goal is to connect physicochemical attributes with delivery performance. In vitro release studies, commonly performed using dialysis-based setups, and ex vivo/in vitro permeation studies, frequently conducted with Franz diffusion cells, are widely used to assess how the formulation behaves under barrier-relevant conditions [17,19]. Although these tests belong partly to performance evaluation rather than strictly to structural characterization, they are often included in the same analytical workflow because they help determine whether the measured CQAs translate into meaningful delivery behavior [17,19]. In recent transferosome studies, such as asiatic acid topical gel and fluconazole transferosomal gel, physicochemical characterization was directly linked to diffusion-cell-based permeation or release analysis to support formulation selection [19,32].

The analytical characterization of transferosomes should be based on methodological complementarity. DLS and zeta potential provide routine colloidal information; TEM, cryo-TEM, and AFM support morphological interpretation; DSC, FTIR, and XRD probe drug–excipient interactions and physical state; scattering methods clarify internal organization; HPLC- or UV-based workflows quantify drug association; and release or permeation studies connect these data with delivery performance. A transferosome formulation is therefore best characterized not by the number of techniques applied, but by the coherence with which orthogonal methods are used to answer structurally and pharmaceutically relevant questions.

## 4. Deformability as the Central Functional Feature

Among the various attributes used to describe transferosomes, deformability occupies a unique position because it is the feature most directly linked to the original rationale of these vesicular systems. While size, charge, morphology, and drug loading define important aspects of formulation quality, deformability is the property historically invoked to explain why transferosomes may behave differently from conventional liposomes under mechanical constraint [11,17,18,62]. For this reason, a critical discussion of transferosomes as drug delivery systems must examine not only how deformability is described, but also how it is defined, measured, compared across studies, and related to biological performance [11,17,18,62].

### 4.1. The Concept of Deformability in Transferosomal Systems

In transferosomal science, deformability refers to the ability of a vesicle to undergo substantial shape adaptation under an external stress while retaining a coherent bilayer structure and a functionally relevant degree of vesicular integrity [11,17,18,62]. This definition is more restrictive than simply describing a membrane as fluid, soft, or elastic. A membrane may be fluid without being sufficiently adaptable to pass through narrow constrictions, and a vesicle may be soft yet structurally unstable to the point of rupture or leakage under stress. Deformability, in the transferosomal sense, therefore implies a specific balance between membrane flexibility and structural cohesion, allowing the vesicle to transiently change shape without immediate disintegration [11,17,18].

This concept is central because transferosomes were originally conceived not merely as lipid vesicles containing surfactants, but as stress-responsive bilayer systems capable of negotiating highly confined pathways more efficiently than conventional liposomes [18,24,62]. The classical mechanistic interpretation proposes that the inclusion of edge activators reduces local packing constraints within the phospholipid bilayer, thereby lowering the energetic penalty associated with bending, squeezing, and shape rearrangement [11,17,18]. Under this view, deformability is not a secondary or accidental consequence of composition, but an intentionally engineered membrane property. It is this feature that gave rise to the original expectation that transferosomes could adapt to narrow intercellular spaces or barrier-associated microenvironments while continuing to act as drug carriers [24,62].

A key conceptual point is that deformability should not be equated with high membrane fluidity alone. Romero and Morilla emphasized that highly deformable vesicles and highly fluid vesicles are related but not identical categories, since the former are defined by their capacity to withstand and respond to stress in a controlled way, whereas the latter may simply possess loosely packed bilayers without necessarily preserving vesicular function under confinement [11,18]. This distinction remains important in the present literature, because transferosomes are sometimes discussed as though any surfactant-containing liposome were automatically “ultradeformable.” In reality, the presence of a bilayer-softening component is not sufficient by itself; deformability is a functional property that emerges only when membrane composition, vesicle architecture, and interfacial conditions combine in a way that allows reversible or quasi-reversible shape adaptation [11,17,18].

Another important distinction is the difference between deformability as a vesicle-level property and permeation enhancement as a formulation outcome. Transferosomal literature has often linked the two very closely, especially in skin delivery, but they are not conceptually identical [17,23,24,62]. A vesicle may be highly deformable yet still fail to improve drug transport if the drug is released too early, if the barrier environment disrupts the vesicle, or if other formulation variables limit tissue interaction. Conversely, a formulation may show improved permeation for reasons that are not directly attributable to deformability, such as surfactant-mediated barrier perturbation, improved thermodynamic activity of the drug, or prolonged surface residence [17,23,24]. This distinction matters because it prevents deformability from being treated as an all-purpose explanation for every favorable transferosome result. Instead, deformability should be regarded as a mechanistically plausible contributor whose functional consequences must be demonstrated rather than assumed [11,17,23].

The concept also has a strong context dependence. Deformability is not expressed in the abstract, but only under particular experimental or biological conditions. The same vesicle may respond differently depending on temperature, osmotic gradient, applied pressure, hydration state, ionic strength, or the geometry of the constriction it encounters [11,17,18]. This is one reason why the original transferosome concept became associated with hydration-gradient-driven movement through the skin under non-occlusive conditions: the vesicle was not thought to be permanently “deformed,” but rather able to deform when the physicochemical environment created the appropriate driving force [24,62]. In this sense, deformability is best understood as a conditional mechanical competence of the vesicle rather than as a fixed material constant [18,24].

Recent reviews continue to frame deformability as the hallmark of transferosomes, but they also increasingly acknowledge that the term is used inconsistently across the literature [17,23,37]. Some studies infer deformability mainly from composition, others from extrusion-based indices, and others from enhanced permeation outcomes, even though these approaches do not necessarily measure the same phenomenon [11,17,23]. This conceptual looseness has contributed to some of the ambiguity surrounding transferosomes and their distinction from elastic liposomes, deformable nanovesicles, and related soft carrier systems [11,17,37]. A more rigorous use of the term, therefore, requires separating three levels of interpretation: composition-level expectations (for example, inclusion of edge activators), measurement-level evidence (for example, deformability indices or mechanical tests), and performance-level consequences (for example, permeation or tissue deposition) [11,17]. Keeping these levels analytically distinct is essential for a clearer understanding of what deformability means and what it does not mean in transferosomal research.

It is also useful to recognize that deformability is fundamentally a systems property. It does not arise from a single excipient, nor can it be predicted solely from the presence of a phospholipid and a surfactant. Instead, it reflects the integrated mechanical behavior of the vesicle as determined by lipid composition, edge activator identity, component ratio, drug incorporation, vesicle size, lamellarity, and environmental conditions [11,17,18]. This systems-level view is particularly important for the interpretation of experimental data, because it explains why formulations with apparently similar ingredient lists may display very different deformation behavior in practice. It also reinforces why deformability must be analyzed in parallel with other critical quality attributes rather than treated as an isolated label attached to the formulation.

From a conceptual standpoint, then, deformability should be regarded as the central functional hypothesis of transferosomes rather than as a self-evident fact. The hypothesis is that a properly engineered vesicle can preserve enough structural integrity while adapting its shape under stress to achieve a delivery advantage across restrictive biological interfaces [11,17,18,24,62]. The following sections, therefore, need to address not only how this property is measured, but also how reliably those measurements reflect the underlying mechanical phenomenon and how convincingly they correlate with biological performance.

### 4.2. Experimental Methods for Deformability Assessment

Experimental assessment of deformability remains one of the most challenging aspects of transferosome characterization because the property itself is dynamic, conditional, and method-dependent. Unlike vesicle size or zeta potential, deformability cannot be captured by a single static descriptor. Instead, it is usually inferred from how the vesicle responds to a defined mechanical constraint, most commonly forced passage through pores smaller than its own diameter [11,17,18,62]. For this reason, experimental methods for deformability assessment do not simply “measure” deformability in an absolute sense; rather, they operationalize it under specific test conditions and generate comparative indices whose meaning depends strongly on the methodology used [11,18,62].

The classical and still most widely cited approach is the extrusion-based deformability assay, in which a vesicle dispersion is driven through a membrane filter with pores smaller than the vesicle diameter under a defined pressure or centrifugal force [11,17,18]. In this setup, deformability is typically expressed through an empirical deformability index that incorporates the amount of suspension extruded over a given time and the ratio between vesicle size after extrusion and membrane pore size [11,18]. The rationale is straightforward: vesicles that can pass more readily through constrictions narrower than their original size, while remaining sufficiently intact to be recovered as vesicles, are considered more deformable than those that resist passage or undergo disruption [11,18,62]. This approach became central to transferosome research because it translates the theoretical concept of stress-adaptive vesicles into a practical comparative test.

Despite its popularity, the extrusion assay should be interpreted with caution. The resulting deformability index is not a universal material constant, but an assay-dependent parameter influenced by membrane pore diameter, applied pressure, extrusion time, lipid concentration, temperature, viscosity of the medium, and the initial size distribution of the vesicles [11,17,18]. Even the choice of whether vesicle size is measured before or after extrusion, and how that size is determined, can alter the final index substantially [11,18]. As a consequence, deformability values reported in different studies are often not directly comparable unless the experimental setup is closely matched. This limitation has been emphasized repeatedly in reviews on carrier deformability and remains one of the main reasons why the literature contains many relative claims of “higher deformability” without a fully harmonized measurement framework [11,17,18].

A closely related variant is the pressure-driven membrane passage assay, in which vesicle transport across a porous membrane is monitored under controlled pressure conditions rather than simply by manual or low-controlled extrusion [11,18,62]. Conceptually, this approach belongs to the same methodological family as the deformability index assay, but it can offer better control over the mechanical driving force and therefore more reproducible stress conditions. Even so, the same interpretative limitations remain: passage through a membrane depends not only on vesicle deformability, but also on vesicle–membrane interactions, possible adsorption to the filter, clogging effects, and any formulation-dependent tendency toward rupture, fusion, or leakage during the test [11,18,62]. Thus, these assays are best understood as comparative permeability-under-stress tests rather than direct, pure measurements of membrane mechanics.

Another important issue is that extrusion-based methods often conflate several phenomena that are experimentally difficult to separate. A vesicle may fail to pass through the membrane because it is mechanically rigid, because it aggregates, because it adheres to the filter, or because it ruptures and loses structural identity during the test [11,18]. Conversely, apparent successful passage does not necessarily prove that the vesicle remained intact throughout deformation; it may indicate partial reassembly after transient disruption, especially in highly fluid systems [11,18]. This is precisely why Romero and Morilla stressed the conceptual distinction between highly deformable vesicles and merely highly fluid vesicles [11,18]. In practical terms, extrusion assays are useful, but they do not by themselves resolve whether the observed behavior reflects true elastic adaptation, partial disintegration, or some combination of both.

Because of these limitations, more recent discussions increasingly treat deformability assessment as a multimethod problem rather than something that can be resolved by a single index [17,37]. In current transferosome literature, deformability is often interpreted together with vesicle size, PDI, morphology, and sometimes release or permeation data, in an attempt to determine whether a formulation that appears highly deformable in extrusion tests also preserves a coherent vesicular organization and meaningful delivery performance [17,37]. This trend is methodologically sensible, because deformability becomes much more informative when the extrusion result is read alongside structural evidence that the vesicles remain vesicular and not merely surfactant-softened dispersions [11,17,18].

Outside the classical extrusion framework, there is growing interest in mechanical and nanostructural techniques that can provide complementary insight into membrane behavior, even if they are not yet standard in transferosome studies. Reviews of soft nanocarrier characterization increasingly point to the value of cryo-TEM for examining whether vesicles retain structural continuity under relevant conditions, and of scattering techniques or surface-sensitive measurements for probing subtle changes in bilayer organization [17,37]. These methods do not yield a conventional deformability index, but they can help clarify whether compositional changes associated with “higher deformability” are also associated with altered lamellarity, membrane thickness, or internal organization. In that sense, they contribute to a more mechanistically grounded interpretation of deformation behavior, even when they do not constitute deformability tests in the narrow classical sense.

**Figure 2:**
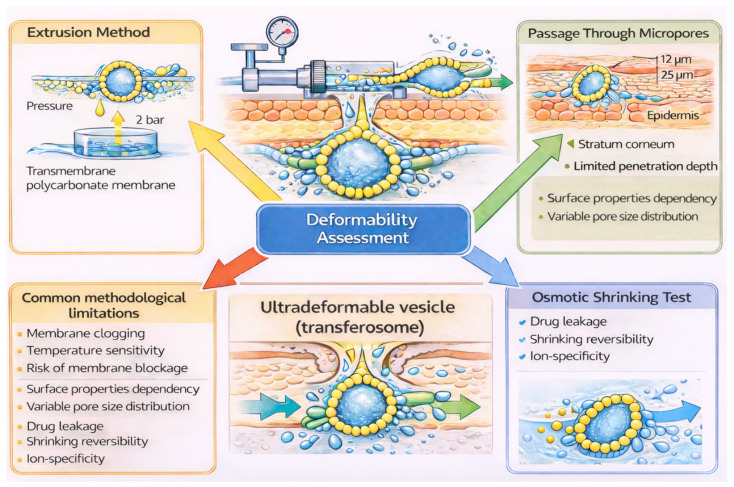
Schematic representation of the main experimental methods used to evaluate transferosome deformability, including extrusion-based assays, pressure-driven membrane passage, and osmotic shrinking tests. Each method provides indirect insight into vesicle flexibility but is influenced by experimental conditions and presents specific limitations.

At present, then, the experimental assessment of transferosome deformability is best viewed as a hierarchy of evidence. Classical extrusion-based assays remain the practical standard because they are simple, inexpensive, and historically embedded in the field [11,17,18,62]. However, their outputs should be interpreted as relative performance indicators under specified conditions, not as absolute measures of a universally defined physical property [11,18]. The strongest experimental case for transferosomal deformability is therefore built when extrusion results are combined with orthogonal characterization showing vesicle integrity, composition-dependent membrane adaptation, and a plausible connection to barrier-relevant behavior. That approach does not eliminate methodological ambiguity, but it does reduce the risk of treating a single deformability index as a sufficient explanation for transferosome performance. A schematic overview of the principal experimental approaches used to assess transferosome deformability and their inherent limitations is provided in Figure 2.

### 4.3. Factors Affecting Measurement Outcomes

The outcome of deformability measurements in transferosomal systems is highly sensitive to experimental conditions, which means that the reported value is not determined solely by intrinsic vesicle properties. Instead, the measured response emerges from an interaction between formulation composition, vesicle population characteristics, assay geometry, applied mechanical stress, and the physicochemical environment in which the test is performed [17,19,37,63]. This is one of the main reasons why deformability data are often difficult to compare across studies, even when similar lipid and surfactant systems are used [19,63]. A proper interpretation of deformability, therefore, requires distinguishing between factors that genuinely alter vesicle mechanics and factors that mainly alter how those mechanics are expressed within a particular assay.

One of the most influential variables is membrane pore size in extrusion-based assays. Because the deformability index is commonly derived from vesicle passage through pores smaller than the initial vesicle diameter, the selected pore size directly determines the level of mechanical challenge imposed on the system [17,19,63]. If the pores are too large relative to the vesicle size, the test may underestimate differences between formulations because even moderately deformable vesicles can pass without substantial stress. Conversely, if the pores are too small, passage may be limited not only by reduced deformability but also by clogging, irreversible disruption, or filter–vesicle interactions [19,63]. This means that deformability values generated with different membrane geometries cannot be treated as directly equivalent unless the test configuration is tightly matched.

A second major determinant is the applied pressure or driving force. In classical deformability assays, vesicle transport across the membrane depends strongly on the magnitude and mode of pressure application, whether pneumatic, centrifugal, or manually imposed [19,63]. Higher pressure may increase the amount of material extruded and thereby raise the calculated deformability index, but this does not necessarily mean that the vesicle is intrinsically more deformable. It may instead indicate that the assay is forcing passage through enhanced mechanical stress, potentially accompanied by vesicle rupture, transient disassembly, or partial reformation after passage [19,63]. For this reason, pressure is not just a procedural parameter; it is part of the definition of the assay itself. Results obtained under different pressure regimes should therefore be interpreted as assay-specific rather than universally comparable descriptors of vesicle mechanics.

Temperature is another critical factor because it directly affects bilayer fluidity, surfactant mobility, phase behavior, and the energetic cost of membrane deformation [17,19,63]. A transferosomal membrane tested near or above the relevant transition region may exhibit greater apparent deformability than the same system tested at lower temperature, simply because the bilayer is more dynamically disordered under those conditions [19,63]. Temperature can also modify the viscosity of the dispersion medium, the permeability of the membrane, and the kinetics of vesicle recovery after stress. As a result, deformability measurements performed at room temperature, physiological temperature, or uncontrolled laboratory temperature may yield substantially different outcomes even for the same formulation. This issue is particularly important in transferosome studies intended for biological application, because the mechanical behavior relevant to storage conditions may not be identical to that relevant to skin or mucosal contact.

The initial vesicle size and size distribution of the formulation also exert a strong influence on measurement results. Deformability assays based on forced passage through pores are inherently sensitive to the relationship between vesicle diameter and membrane pore diameter [19,63]. A formulation with a broader size distribution may appear less deformable simply because a fraction of the vesicle population is larger and more prone to obstruction or rupture, whereas a narrower and smaller population may pass more readily, even if membrane mechanics are not fundamentally different [19]. This means that deformability results should not be interpreted independently of DLS- or microscopy-based population data. In practice, apparent improvements in deformability may sometimes reflect changes in size distribution or post-processing history rather than changes in bilayer mechanics alone.

The lipid concentration and overall vesicle loading of the test suspension can also affect the measured outcome. At higher concentrations, vesicle–vesicle interactions, crowding at the membrane surface, and filter clogging may reduce apparent passage efficiency, leading to lower deformability indices even when the membrane composition is unchanged [19,63]. In contrast, highly diluted dispersions may pass more easily and appear more deformable under identical pressure conditions. This effect becomes particularly relevant in soft and surfactant-rich systems, where mechanical interactions among vesicles may influence how stress is distributed during the assay. Consequently, concentration should be treated as a controlled analytical variable, not simply as a convenience of sample preparation.

Medium composition is another major source of variation. The hydration medium influences osmotic balance, ionic strength, pH, surfactant ionization, and electrostatic interactions, all of which can alter vesicle packing and response to stress [19,63]. In transferosomes containing bile salts or ionic excipients, small changes in buffer composition may significantly affect bilayer organization and filter passage. Even in nominally non-ionic systems, changes in ionic strength or osmolarity may modify hydration forces and vesicle integrity during measurement [19]. This is why deformability values obtained in distilled water, buffer, saline, or biologically relevant simulated media should not be assumed to reflect the same mechanical state. The assay medium is part of the measurement context and may substantially influence the apparent outcome.

The composition of the membrane itself obviously remains central, but even here, the effect on measurement outcome is not always straightforward. Phospholipid identity, edge activator type, surfactant concentration, cholesterol content, and incorporated drug all alter bilayer packing and therefore affect deformation behavior [17,19,37,63]. However, what is measured experimentally is not simply a direct readout of composition. For example, increasing surfactant content may enhance apparent deformability up to an optimal point, after which further addition may instead promote leakage, instability, or even structural disintegration during the test [19,63]. In such cases, a lower measured deformability index may reflect loss of vesicle integrity rather than a true increase in membrane rigidity. This nonlinearity is one reason why formulation variables and measurement variables must always be interpreted together.

A further complication arises from post-assay vesicle recovery and the way the deformability index is calculated. Many formulations are characterized using size values measured after extrusion, yet the resulting vesicle population may not correspond exactly to the pre-assay population that experienced deformation [19,63]. If vesicle disruption, resizing, or reassembly occurs during passage, the measured post-extrusion diameter may partly reflect the outcome of stress exposure rather than the original deformability of intact vesicles. The calculated deformability index, therefore, contains embedded assumptions about vesicle continuity that are not always independently verified. This is one reason why orthogonal structural characterization, such as microscopy before and after the test, can add important context to deformability measurements.

These sources of variability explain why measurement outcomes should be interpreted as condition-dependent comparative indicators rather than absolute mechanical constants. The most informative deformability data are usually obtained when pore size, pressure, temperature, concentration, medium composition, and vesicle size distribution are all clearly reported and held under controlled conditions across the formulations being compared [17,19,37,63]. When those variables are not harmonized, differences in the reported deformability index may reflect differences in assay setup as much as differences in vesicle behavior. For a critical review of transferosomes, this point is essential: methodological transparency is not a minor analytical detail but a prerequisite for meaningful interpretation of deformability claims.

### 4.4. Limitations and Reproducibility Issues

One of the most persistent weaknesses in transferosome research is that deformability is frequently presented as a defining advantage, yet the experimental evidence supporting that claim is often difficult to reproduce or compare across studies [47,64,65,66]. The problem is not necessarily that deformability assays are useless, but that they are commonly performed under poorly harmonized conditions and interpreted without sufficient analytical context [47,64,65,66]. In practice, differences in pore size, applied force, lipid concentration, temperature, buffer composition, vesicle population, and post-assay sizing can all influence the reported outcome, which means that a deformability value obtained in one laboratory may not be directly comparable to a value reported elsewhere [47,64,65,66]. This broader issue of insufficient methodological standardization is not unique to transferosomes; it has been recognized across lipid nanocarrier characterization and nanomedicine more generally [64,65,66,67,68,69].

A second limitation is the tendency to treat deformability as if it were a single, self-explanatory parameter [47,64,66]. In many studies, a calculated deformability index is reported without sufficient detail on the measurement setup, the way vesicles were recovered after passage, or whether vesicle integrity was independently confirmed before and after the assay [47,64,66]. Under those conditions, it becomes difficult to know whether the result reflects elastic adaptation, partial disruption, filter interactions, population reshaping, or a combination of these processes [47,64,66]. Reviews on analytical characterization of liposomes and lipid nanoparticles have repeatedly stressed that no isolated measurement should be overinterpreted when the system is structurally complex or mechanically soft [47,64,66]. In transferosomes, this caveat is especially important because the vesicles are intentionally designed to be compositionally labile and stress-responsive [47,66].

Reproducibility is further compromised by incomplete reporting [65,67,68,69]. Many publications provide nominal composition and a final deformability value, but omit key experimental details such as membrane material, pore geometry, pressure regime, equilibration conditions, sample dilution, replicate structure, or the exact formula used to calculate the index. Without those elements, the assay cannot be meaningfully reproduced, and interstudy comparison becomes largely qualitative [65,67,68,69]. More generally, the nanomedicine field has already identified poor reporting of preparation and characterization protocols as a major source of irreproducibility, leading to the development of reporting frameworks such as MIRIBEL and broader calls for transparent, use-specific analytical standards [67,68,69]. Those concerns apply directly to transferosome deformability studies, where seemingly small procedural differences can substantially change the measured outcome [65,67,68,69].

Another recurring issue is the weak separation between measurement-level evidence and performance-level inference [47,66,70]. In the transferosome literature, enhanced permeation, improved deposition, or better therapeutic response is sometimes taken as indirect confirmation of superior deformability, even when deformability itself has not been rigorously or comparably measured. This creates a circular logic in which favorable biological performance is used to validate a mechanical property that was only loosely defined in the first place. From a quality perspective, this is problematic. Regulatory and characterization-oriented reviews on liposomes increasingly emphasize the need to connect critical quality attributes with analytically robust methods and with clearly justified claims, especially when those claims are meant to support product differentiation or translational value [66,70]. The same standard should apply to transferosomes.

There is also a scale and manufacturing dimension to the reproducibility problem [71,72,73]. A deformability result obtained from a small laboratory batch prepared under manually controlled conditions may not survive scale-up, process transfer, or routine production. Batch-to-batch variability in lipid hydration, solvent removal, post-processing, and vesicle downsizing can affect size distribution, lamellarity, and membrane composition, all of which, in turn, alter measured deformation behavior [71,72,73]. Quality-by-design discussions in lipid nanocarriers have highlighted exactly this point: robust product development requires identifying which formulation and process variables control the critical outputs, and then measuring those outputs with methods that remain meaningful beyond exploratory bench-scale work [71,72,73]. Transferosome research would benefit from adopting that same discipline more consistently.

These limitations do not imply that deformability should be abandoned as a concept, but they do show that it must be handled with greater methodological restraint. For transferosome studies to become more reproducible, deformability assays need clearer experimental reporting, better analytical validation, and more frequent use of orthogonal evidence showing what happened to vesicle structure during or after the test. A stronger framework would also distinguish more explicitly between: (i) compositional features expected to promote deformability, (ii) assay-specific measurements of deformation under defined stress, and (iii) downstream biological consequences that may or may not be caused primarily by that property. That separation would improve both interpretability and reproducibility, and would place transferosome research on firmer analytical ground.

### 4.5. Relationship Between Deformability and Biological Performance

The relationship between transferosomal deformability and biological performance is one of the most important, yet also one of the most frequently oversimplified, questions in this field. In principle, deformability is expected to improve biological performance because a vesicle that can adapt its shape under stress should be better equipped to negotiate restrictive biological interfaces, particularly the stratum corneum and associated intercellular pathways [17,25]. This expectation is deeply embedded in the transferosome concept and continues to be repeated in recent reviews and primary studies [17,37]. However, the available evidence indicates that the relationship is suggestive rather than strictly linear: higher deformability often accompanies improved permeation or tissue deposition, but it does not guarantee it, nor does it fully explain biological performance on its own [17,25,37].

A number of primary studies do support a positive association between deformability and skin delivery outcomes. In a widely cited study on diclofenac sodium-loaded ultradeformable vesicles, El Zaafarany et al. showed that changes in edge activator type and surface charge altered vesicle deformability and were accompanied by changes in skin delivery behavior, supporting the view that membrane adaptability contributes to enhanced transdermal performance [25]. Likewise, Lin et al. reported that charged ultradeformable lipid vesicles for imperatorin delivery exhibited improved skin permeation efficiency, and the authors linked this outcome to both deformability and optimized vesicle–skin interactions [74]. These findings are consistent with the classical view that vesicles capable of mechanical adaptation may enhance barrier crossing more effectively than conventional liposomes [25,74].

Recent transferosome studies continue to reinforce this association, but they also show that deformability is only part of a broader performance equation. In hydrocortisone-loaded transferosomes, Abdelwahd and Abdul Rasool identified an optimized formulation that combined suitable elasticity, sustained release, and markedly enhanced ex vivo skin permeation relative to the control, suggesting that deformability can contribute meaningfully to biological performance when aligned with appropriate release behavior and vesicle composition [42]. Similarly, in asiatic acid-loaded transferosomal gels, Opatha et al. reported significantly improved permeation and flux relative to non-transferosomal controls, but the superiority of the formulation depended not only on vesicular design but also on the specific edge activator used and on the final gel vehicle [19]. These studies illustrate that deformability can be functionally beneficial, but its contribution is expressed through a system that also includes drug release kinetics, formulation rheology, and barrier interaction [19,42].

The same point emerges in more recent work on phloretin transferosomes. Wang et al. developed a phloretin transfersome gel with favorable physicochemical properties and demonstrated increased skin penetration together with improved in vivo pharmacokinetic performance compared with a non-transferosomal comparator [61]. Although such results are entirely compatible with the proposed role of deformability, they also make clear that improved biological performance cannot be attributed to deformability alone. In that study, membrane composition, drug solubilization, vesicle stability, and dosage-form design were all part of the formulation logic leading to better delivery [61]. In other words, deformability may enable performance, but it acts within a multivariable formulation context rather than as an isolated causal lever.

This distinction is important because biological performance depends on more than the ability of a vesicle to squeeze through a mechanical constraint. A transferosomal system must also retain a sufficient fraction of the drug, remain physically coherent long enough to interact productively with the barrier, release the payload at an appropriate rate, and behave favorably in the actual application environment [17,37]. If any of these conditions are not met, a highly deformable vesicle may fail to translate its mechanical advantage into better biological output. A surfactant-rich formulation, for example, may show a high deformability index but also suffer from premature drug leakage or instability, which would reduce the effective amount of drug delivered to the target site [17,25]. This helps explain why some studies report strong deformability together with only moderate permeation improvement, whereas others achieve favorable biological results through a more balanced combination of deformability, drug loading, and formulation stability [19,42,61].

A second reason why the correlation is not absolute is that improved biological performance may arise through mechanisms that are only partly related to deformability. Edge activators and other amphiphilic excipients can alter the thermodynamic activity of the drug, fluidize stratum corneum lipids, modify vesicle–barrier interactions, or increase surface residence time, all of which may improve drug transport even if vesicle deformation is not the dominant mechanism [17,25,37]. Thus, when a transferosomal formulation outperforms a conventional control, the result may reflect a composite effect of membrane adaptability, surfactant-mediated barrier modulation, altered release kinetics, and drug-specific partitioning behavior [17,25]. A critical reading of the literature, therefore, suggests that deformability is best viewed as an important contributor to biological performance, but not as a sufficient standalone explanation for it [17,25,37].

Broader vesicular delivery literature supports this more cautious interpretation. Comparative work on diclofenac-loaded lipid vesicles has shown that vesicle architecture, drug localization, and skin interaction collectively influence transdermal delivery outcomes, and that favorable vesicular properties do not automatically translate into maximum permeation [75,76]. In the case of transferosomes, this means that a high deformability index should be interpreted as part of a performance profile, not as a surrogate endpoint that replaces direct biological evaluation. The most persuasive evidence of functional relevance arises when deformability data are accompanied by coherent release, permeation, deposition, and efficacy findings under well-controlled experimental conditions [19,42,61,75,76].

Recent reviews increasingly reflect this position. Rather than presenting deformability as a universally predictive marker, they tend to frame it as a central but context-dependent attribute whose biological importance depends on the barrier being targeted, the drug being delivered, and the formulation environment in which the vesicle operates [17,37]. This interpretation is more consistent with the current evidence base and also more useful from a formulation science perspective. It suggests that the key question is not whether deformability matters in general, but under which compositional, structural, and biological conditions it matters enough to generate a reproducible delivery advantage [17,37].

For the purposes of transferosome research, the most defensible conclusion is that deformability is biologically relevant, but only when interpreted within a broader network of formulation properties. Claims about enhanced delivery should therefore be supported not just by deformability indices, but by integrated evidence linking vesicle mechanics to drug retention, barrier interaction, permeation behavior, and, where possible, therapeutic outcome [17,19,25,37,42,61,74,75,76]. That standard is more demanding than the traditional narrative of “higher deformability equals better delivery,” but it offers a more realistic and analytically robust basis for understanding what transferosomes actually contribute as drug delivery systems.

## 5. Drug Release and Interaction with Biological Barriers

Once transferosomes have been defined in terms of composition, structure, and deformability, the next critical question is how they handle the drug itself during delivery. Their pharmaceutical value depends not only on whether vesicles can be formed and characterized, but also on whether the incorporated drug remains associated with the carrier long enough to reach the barrier, is released at a suitable rate, and interacts with the biological interface in a way that improves local deposition or trans-barrier transport [1,2,3,17,19,37]. This section, therefore, focuses on the relationship between vesicular organization and drug fate, with particular emphasis on incorporation, retention, release behavior, and the consequences of these processes for biological barrier interaction.

### 5.1. Drug Incorporation, Retention, and Release Behavior

Drug incorporation in transferosomal systems is governed by the same general principles that apply to other phospholipid vesicles, but the presence of edge activators adds an additional layer of complexity because it modifies bilayer packing, interfacial polarity, and membrane permeability [17,19]. In practical terms, hydrophilic compounds tend to partition preferentially into the aqueous core and interlamellar aqueous domains, whereas lipophilic drugs are more likely to associate with the hydrophobic bilayer. Amphiphilic compounds may distribute between both regions depending on ionization state, membrane composition, and the characteristics of the hydration medium [17,19,37]. This partitioning behavior is central because it determines not only apparent encapsulation efficiency, but also how strongly the payload is retained within the vesicle and how it will be released upon contact with the target barrier [17,19].

Retention is particularly important in transferosomes because these carriers are intentionally designed with membranes that are more adaptable and, at the same time, potentially more permeable than those of conventional liposomes [17,19]. A formulation may show excellent initial drug association yet still be pharmaceutically weak if the drug redistributes rapidly into the external medium during storage, dilution, or biological exposure. For that reason, the relevant question is not simply whether the drug can be incorporated, but whether it remains associated with the vesicle long enough to support the intended delivery mechanism [17,37]. In transferosomal systems, retention is affected by drug lipophilicity, phospholipid composition, edge activator identity, cholesterol content when present, and the physicochemical environment surrounding the vesicle [17,19,37]. A highly lipophilic compound may be retained strongly in the bilayer, whereas a water-soluble or weakly bound payload may leak more readily if membrane softness is increased beyond an optimal range [17,19,37].

Release behavior in transferosomes is therefore best understood as a consequence of the balance between drug–membrane affinity and membrane permeability. If the drug interacts too strongly with the bilayer, release may be excessively slow, which can favor localized deposition but limit effective partitioning into tissue. If retention is too weak, the vesicle may function mainly as a short-lived solubilization system rather than as a carrier capable of transporting the drug in a controlled way [17,19]. This balance is especially relevant in transferosomes because edge activators can simultaneously promote deformability and increase the likelihood of drug escape, so the same compositional change that improves barrier negotiation may also alter release kinetics in an unfavorable direction [17,19]. For this reason, release behavior should not be interpreted independently of deformability, stability, and barrier interaction. It is one of the key points where these formulation attributes converge.

A recurring observation in the transferosome literature is that these vesicles often produce a slower and more sustained release profile than drug solutions or conventional dispersions, although the extent of this effect is highly formulation-dependent [16,19,21,33,61]. In the classical diclofenac study by Cevc and Blume, ultradeformable carriers were reported to facilitate highly efficient topical and transdermal delivery, a finding that helped establish the idea that vesicle composition could modulate both retention and release in a way that enhanced biological performance [16]. More recent studies support the same general principle, but with a clearer emphasis on formulation-specific behavior. In curcumin-loaded transferosomes for ocular delivery, Barbalho et al. reported extremely high drug association together with minimal initial burst release and prolonged release behavior, which is consistent with the strong bilayer affinity of curcumin and the ability of the vesicle to function as a sustained ocular delivery platform [21]. In phloretin transferosomes, Wang et al. likewise observed a release pattern that was slower and more controlled than that of the free drug, while also reporting improved skin delivery and in vivo performance [61]. These examples indicate that transferosomes can support sustained release, but that this behavior emerges from the compatibility between the drug and the membrane rather than from deformability alone.

The same conclusion emerges from more application-oriented gel systems. In asiatic acid-loaded transferosomal gels, Opatha et al. demonstrated that entrapment into transferosomes markedly improved permeation compared with non-transferosomal controls, yet the outcome depended strongly on the nature of the edge activator and on the final semisolid vehicle [33]. In hydrocortisone transferosomes, Abdelwahd and Abdul Rasool reported a formulation that combined sustained release with enhanced ex vivo skin permeation, again suggesting that favorable delivery performance resulted from a coordinated relationship among drug incorporation, release control, and barrier interaction rather than from any single parameter in isolation [42]. Similarly, optimized fluconazole transferosomal gels have been described as systems in which drug compatibility, controlled release, and improved topical performance were addressed simultaneously during formulation development [32]. What these studies collectively show is that release behavior becomes pharmaceutically meaningful only when interpreted alongside retention and biological response.

Another important point is that in transferosomal systems, release behavior measured in vitro is not always equivalent to release behavior at the biological barrier. Dialysis-based assays and diffusion-cell studies are useful for comparing formulations, but they simplify the environment in which release actually occurs. At a real biological interface, drug release is influenced by vesicle adsorption, hydration gradients, local lipid interactions, pH, ionic strength, tear or skin fluid composition, and the possibility of vesicle restructuring after contact with the barrier [17,21,33]. This is why formulations with apparently similar in vitro release profiles may still behave differently in permeation or tissue-deposition experiments. In curcumin ocular transferosomes, for example, formulation performance varied across ocular tissues despite shared vesicular design principles, underscoring that release and penetration are jointly shaped by the local barrier environment [21]. The same logic applies to skin delivery, where vesicle retention at the surface, interaction with stratum corneum lipids, and local partitioning of the drug all influence the final pharmacological outcome [17,33,61].

From a formulation perspective, the most useful interpretation is to treat incorporation, retention, and release as a linked continuum rather than as separate endpoints. Drug incorporation determines where the payload resides in the vesicle; retention determines whether that association persists long enough to matter; and release behavior determines how the drug becomes available at the barrier or within the tissue [17,19,37]. A transferosomal formulation is most likely to be effective when these three elements are aligned with the intended route of administration and therapeutic objective. For local topical therapy, prolonged retention and slower release may be desirable if they enhance depot formation in superficial tissues. For transdermal or ocular delivery, the formulation may need to retain the drug long enough to reach the relevant interface, but not so strongly that release into the target tissue becomes rate-limiting [17,21,33,42,61]. This is one of the reasons why apparently similar transferosomal compositions can produce very different delivery outcomes when loaded with different drugs.

A critical implication of this framework is that neither high incorporation nor slow release should be treated as inherently superior. A high-loading formulation may still perform poorly if release is excessively restricted, while a formulation with moderate loading may be more effective if it shows better retention during application and more suitable release at the barrier [17,19]. The relevant criterion is therefore not the magnitude of any individual parameter, but whether the formulation establishes a coherent relationship among drug association, vesicle stability, release kinetics, and biological interaction. In transferosome research, this integrated view offers a more realistic basis for interpretation than simply assuming that higher entrapment or slower release automatically translates into better delivery.

### 5.2. Interaction with the Skin Barrier

The interaction of transferosomes with the skin barrier is central to their pharmaceutical rationale because the stratum corneum remains the principal obstacle to dermal and transdermal delivery. This outermost layer is composed of corneocytes embedded in a highly ordered lipid matrix and is exceptionally effective at limiting the penetration of most hydrophilic molecules, macromolecules, and many poorly partitioning actives [24,77]. Transferosomes were originally proposed as vesicular systems able to engage this barrier differently from conventional liposomes by combining vesicle-mediated drug carriage with a membrane architecture capable of adapting to highly confined intercellular environments [23,24].

A classical mechanistic interpretation is that transferosomes interact with the skin under non-occlusive conditions through a hydration-gradient-driven process. In this view, water activity differences between the skin surface and deeper viable layers create a driving force that promotes vesicle movement toward more hydrated regions, while the deformable membrane facilitates passage through narrow intercellular pathways [24]. This mechanism has been highly influential in the transferosome field because it provides a conceptual explanation for why vesicles larger than the nominal dimensions of intercellular spaces might still contribute to enhanced drug delivery. At the same time, later reviews have made clear that this explanation should be treated as a working model rather than as a universally proven mechanism for every formulation and every skin condition [77,78].

Current evidence suggests that transferosome–skin interaction is better understood as a multifactorial process rather than as the consequence of deformability alone. Several elements may contribute simultaneously: partial vesicle penetration into superficial or deeper layers, close interfacial contact with the stratum corneum, lipid exchange between vesicles and skin lipids, local fluidization or perturbation of barrier lipids by edge activators, and drug release at or within the barrier, followed by partitioning into viable tissue [15,77,78]. This broader interpretation is consistent with comparative work on ultradeformable vesicles, where differences in skin delivery could not be explained solely by the presence or absence of a transferosome label, but instead depended on the combined effects of vesicle composition, drug polarity, and barrier interaction [15].

The role of edge activators is particularly important in this context. These surfactants do not simply soften the membrane; they may also influence how the vesicle interacts with the skin lipid matrix, alter interfacial tension, and contribute to local changes in barrier organization. In a widely cited study, El Zaafarany et al. showed that edge activator type and vesicle surface charge significantly affected deformability and skin delivery behavior, indicating that the transferosome–skin relationship depends strongly on membrane composition rather than on deformability as an isolated abstract property [25]. This finding remains important because it links barrier interaction to controllable formulation variables and helps explain why formulations with apparently similar vesicle size or morphology may behave differently in permeation studies.

Another key point is that transferosomes do not necessarily need to traverse the entire stratum corneum intact in order to improve biological performance. Reviews of liposomal and ultradeformable systems increasingly support the idea that enhanced skin delivery may arise from a combination of phenomena, including vesicle adsorption, localized drug release, improved thermodynamic activity of the drug, and modification of the superficial lipid environment [77,78,79]. In that sense, the skin barrier should not be viewed as a passive sieve through which transferosomes either pass or fail to pass. Rather, it is an active interfacial environment in which vesicles may partially penetrate, reorganize, fuse, deposit components, or release drugs in a spatially heterogeneous way [78,79]. This interpretation is particularly useful because it accommodates the diversity of experimental outcomes reported across different drugs, vehicles, and skin models.

Recent primary studies remain consistent with this more nuanced model. In asiatic acid-loaded transferosomal gels, Opatha et al. reported improved in vitro permeation and flux relative to non-transferosomal controls, supporting the conclusion that transferosomes can enhance interaction with the skin barrier in a way that facilitates drug transport [33]. Likewise, Wang et al. showed that phloretin-loaded transferosomes improved transdermal delivery and in vivo pharmacokinetic behavior, findings that imply a favorable interplay among vesicle composition, drug retention, and skin interaction rather than a simple one-step penetration mechanism [61]. These studies are important because they reinforce the biological relevance of transferosome–skin interaction while also showing that the final outcome depends on the full delivery system, including the dosage form and the physicochemical properties of the incorporated drug.

Skin condition itself adds another layer of complexity. Barrier interaction may differ substantially depending on hydration state, anatomical site, species, integrity of the stratum corneum, and whether the formulation is tested on intact skin, compromised skin, or artificial membranes [77,79]. This is one reason why mechanistic conclusions drawn from ex vivo permeation models should be interpreted carefully when extrapolating to clinical performance. Transferosome–skin interaction is therefore not a fixed property of the vesicle alone, but a context-dependent process shaped jointly by vesicle mechanics, formulation environment, drug properties, and the physiological state of the barrier [77,78,79].

From a formulation perspective, the most defensible view is that transferosomes enhance skin delivery by establishing a more favorable vesicle–barrier interface. Deformability remains relevant because it may allow better adaptation to confined spaces and barrier heterogeneity, but the actual pharmacological benefit emerges only when this property is coupled with adequate drug retention, suitable release kinetics, and productive interaction with stratum corneum lipids [15,25,33,61,77,78]. For that reason, claims about superior skin penetration should ideally be supported by convergent evidence from vesicle characterization, permeation studies, deposition data, and mechanistic interpretation rather than by deformability indices alone.

### 5.3. Interaction with Mucosal and Ocular Barriers

Compared with the skin, mucosal and ocular barriers present a different balance of opportunity and constraint for transferosomal delivery. These interfaces are generally more hydrated and, in many cases, less structurally restrictive than the stratum corneum, but they are also protected by rapid clearance mechanisms, including mucus turnover, tear drainage, and blinking, which can markedly reduce formulation residence time and limit effective drug absorption [7,8,80]. For this reason, transferosome performance at mucosal and ocular surfaces cannot be inferred directly from transdermal behavior. Instead, it depends on how vesicle deformability, interfacial compatibility, drug retention, and formulation residence interact with the physiology of each barrier [8,80,81].

At mucosal sites, the main challenge is not simply crossing an epithelial layer, but maintaining productive contact with a hydrated and frequently mucus-covered surface long enough to permit drug absorption. Mucus acts as a dynamic barrier with steric, adhesive, and physicochemical filtering properties, while the underlying epithelium further regulates diffusion and transport [7,8]. In this setting, transferosomes are attractive because their soft bilayer structure may improve adaptation to the interfacial environment, and because they can be incorporated into secondary dosage forms such as mucoadhesive or in situ gelling systems to extend residence time [80,81,82,83]. This is particularly relevant for nasal and buccal delivery, where rapid clearance often limits the performance of conventional formulations.

The nasal route is one of the most investigated mucosal applications of transferosomes. Here, the potential advantages include improved local absorption, avoidance of hepatic first-pass metabolism, and, in selected cases, the possibility of nose-to-brain delivery [80,81,82,83]. Salem et al. developed a nanosized transferosome-based intranasal in situ gel for resveratrol and reported enhanced bioavailability relative to control formulations, supporting the idea that a deformable vesicular system combined with a retention-promoting vehicle can perform effectively at the nasal mucosa [81]. Aboud et al. similarly showed that carvedilol-loaded nanotransfersomes administered intranasally improved in vivo delivery behavior compared with conventional administration [83]. More recently, ElSayed et al. reported hyaluronic-acid-coated transfersomes for intranasal delivery of donepezil, further reinforcing the importance of combining vesicle deformability with mucoadhesion and formulation engineering to counter rapid mucociliary clearance [82].

The buccal route illustrates a related but distinct scenario. Buccal mucosa offers convenient access and avoids gastrointestinal degradation and hepatic first-pass metabolism, yet successful delivery still requires sufficient residence at a moist, mechanically active surface and adequate transport across a stratified epithelium [8,84]. In this context, transferosomes have been explored as carriers capable of improving mucosal contact while sustaining drug release. Elkomy et al. developed a loratadine buccal transferosomal gel and demonstrated enhanced ex vivo permeation together with favorable pharmacokinetic performance in human volunteers relative to the oral product [84]. These findings suggest that at buccal barriers, the practical benefit of transferosomes is linked less to a simple penetration narrative than to the combined effects of mucosal adaptation, retention, and controlled drug release.

Ocular barriers impose another distinct set of constraints. Topically applied ocular formulations must contend with tear turnover, blinking, nasolacrimal drainage, corneal epithelial tight junctions, conjunctival clearance, and limited contact time, all of which contribute to the low bioavailability of conventional eye drops [21,80]. Transferosomes have attracted growing attention in this context because their deformable membrane may improve surface adaptation and tissue penetration while maintaining the advantages of topical administration [21,49,51]. Importantly, however, ocular performance depends strongly on how the vesicles behave in the tear environment rather than only on how they are characterized in purified media.

Recent ocular transferosome studies provide some of the clearest evidence for barrier-specific behavior. Barbalho et al. developed curcumin-loaded transferosomes for topical ocular delivery and found enhanced penetration into several ocular tissues, with different penetration patterns across tissues, indicating that barrier interaction was not uniform throughout the eye [21]. Bhujbal et al. later formulated tonabersat transferosomes for ocular delivery and showed that the system could improve penetration-related performance while maintaining acceptable physicochemical characteristics [51]. In a related study, the same group demonstrated that tear-fluid properties significantly altered transferosome size, polydispersity, zeta potential, mucoadhesion, and ocular penetration behavior, highlighting that ocular transferosome performance is highly sensitive to the local physicochemical environment [49].

These results support a more realistic interpretation of transferosome action at mucosal and ocular barriers. Their pharmaceutical value is not simply that they “penetrate better,” but that they can establish a more favorable interface with hydrated, clearance-prone biological surfaces. At mucosal sites, this may translate into improved residence and epithelial contact when combined with a mucoadhesive design [8,81,82,83,84]. At the ocular surface, it may mean better retention in the tear film and improved partitioning into corneal or conjunctival tissues under topical administration [21,49,51]. The specific outcome depends strongly on the target barrier, the local fluid environment, the incorporated drug, and the final dosage form.

For that reason, transferosome interaction with mucosal and ocular barriers should be understood as a barrier-specific formulation phenomenon rather than as a direct extension of the skin-delivery paradigm. A mechanically adaptable vesicle may be advantageous in these environments, but its practical performance emerges only when deformability is aligned with sufficient drug retention, compatibility with local fluids, suitable release behavior, and enough residence time to overcome rapid clearance [8,21,49,51,80,81,82,83,84].

### 5.4. Intact Vesicle Penetration, Prior Drug Release, and the Factors Governing Tissue Deposition and Permeation

One of the longest-standing questions in transferosome research is whether these vesicles cross biological barriers as intact carriers or whether their main contribution lies in releasing the drug before or during interaction with the barrier. This issue is particularly relevant for skin delivery, where the original transferosome concept was strongly associated with the idea of stress-adaptable vesicles moving along hydration gradients and traversing narrow intercellular pathways more efficiently than conventional liposomes [16,24]. That mechanistic hypothesis has been highly influential and remains an important conceptual foundation of the field. However, the subsequent literature has made clear that the experimental evidence is more nuanced than a simple “intact penetration” model would suggest [16,77,78].

Current understanding supports a broader interpretation in which transferosome-mediated delivery may involve several non-mutually exclusive processes: partial or superficial vesicle penetration, close adsorption to the barrier surface, local lipid exchange with the barrier, surfactant-assisted modulation of barrier structure, drug release at or within the barrier, and subsequent diffusion of the liberated drug into deeper tissues [14,16,77,78]. Under this view, intact vesicle penetration is not necessarily an all-or-none event, and it may not be the dominant mechanism for every formulation or every drug. Instead, the relative importance of intact vesicle transport versus prior drug release is expected to depend on formulation composition, drug–membrane affinity, vesicle stability, barrier physiology, and the surrounding microenvironment [14,16,77,78].

To make these mechanistic alternatives explicit, Table 3 summarizes the main interpretative models proposed for transferosome-mediated delivery and the principal limitations associated with each one.

This comparison indicates that no single mechanism can be assumed to explain all transferosome-mediated delivery outcomes. The intact-vesicle model remains central to the historical identity of transferosomes, but current evidence supports a more plural interpretation in which vesicle integrity, drug release, surfactant-mediated barrier modulation, and local interfacial adaptation may contribute to different extents depending on the formulation and the target barrier. Therefore, mechanistic claims should be made at the level of a specific vesicle–drug–barrier system rather than generalized to transferosomes as a whole [14,15,16,25,33,61,77,78].

This distinction matters because tissue deposition and permeation are not synonymous endpoints. A formulation may produce high tissue deposition by retaining the drug within superficial or intermediate barrier layers, which can be advantageous for local dermatological therapy, while a different formulation may favor trans-barrier permeation into deeper tissues or systemic circulation [16,25,77]. In transferosome research, these outcomes are often discussed together, but they are governed by partly different factors. Vesicles that retain the drug strongly and interact favorably with the outer barrier may enhance local accumulation without necessarily maximizing trans-barrier flux, whereas vesicles with more rapid release or different membrane composition may promote deeper diffusion of the drug after contact with the barrier [16,25,77].

Several studies support the idea that the biological outcome depends on this balance rather than on a single penetration mechanism. In a classical study on diclofenac-loaded ultradeformable vesicles, Cevc and Blume reported highly efficient topical and transdermal delivery and framed the result in terms of the special transport behavior of transferosomes [16]. Yet later comparative and mechanistic work has shown that enhanced delivery can also arise from barrier perturbation, interfacial adaptation, and local drug release rather than only from intact vesicle passage [14,77,78]. El Zaafarany et al. demonstrated that edge activator type and surface charge significantly influenced both deformability and skin delivery, indicating that permeation and deposition are strongly tied to membrane composition and vesicle–barrier interaction, not simply to the existence of a deformable vesicle [25]. This is important because it suggests that the route by which the drug reaches the tissue may vary between formulations, even when both are labeled as transferosomes.

A similar conclusion emerges from more recent topical studies. In asiatic acid-loaded transferosomal gels, Opatha et al. showed that transferosome incorporation significantly improved permeation relative to non-transferosomal controls, but the magnitude of the effect depended strongly on edge activator selection and the final gel system [33]. In phloretin transferosomes, Wang et al. reported enhanced transdermal delivery and improved in vivo performance, yet the results were clearly linked to the combined effects of vesicle composition, sustained drug association, and release behavior rather than to deformability alone [61]. These examples indicate that the decisive question is not simply whether transferosomes penetrate intact, but how the formulation establishes an effective sequence of retention, release, and barrier interaction that leads to useful drug deposition or permeation.

Drug properties are among the strongest factors governing this outcome. Lipophilic compounds that partition strongly into the bilayer may remain vesicle-associated for longer periods and therefore be more likely to participate in vesicle-mediated interfacial transport or localized depot formation. By contrast, hydrophilic or weakly retained drugs may redistribute more rapidly into the surrounding medium, making prior or concurrent drug release a more important contributor to the final transport profile [77,78]. Amphiphilic and ionizable compounds are especially sensitive to local pH, ionic strength, and membrane composition, which can alter where the drug resides in the vesicle and when it becomes available for partition into tissue [78]. As a result, the same transferosomal composition may favor deposition for one drug and permeation for another.

Membrane composition is equally decisive. Phospholipid identity, edge activator type, surfactant concentration, cholesterol content, and surface charge modifiers all influence not only deformability, but also membrane permeability, drug retention, and barrier affinity [14,25,33,77,78]. A formulation with strong membrane softening may favor closer interfacial adaptation and faster release, while a more cohesive vesicle may preserve drug association longer and promote sustained local deposition. This is why changing the edge activator can alter not only the measured deformability index, but also whether the formulation behaves primarily as a penetration enhancer, a local drug depot, or a trans-barrier carrier [25,33]. The consequence is that tissue deposition and permeation should be interpreted as emergent properties of the full vesicle–drug–barrier system rather than as direct outputs of a single formulation variable.

Barrier condition and experimental model introduce another major source of variation. Hydration state, anatomical site, epithelial thickness, lipid composition, and species differences can all alter how a transferosomal formulation behaves at the interface [14,15,77]. Ex vivo porcine or rodent skin, reconstructed membranes, mucosal tissues, and ocular surfaces do not present identical transport environments, and, therefore, the relative roles of intact vesicle penetration and prior drug release may differ accordingly [14,77]. This is one reason why permeation results are often not directly comparable across studies and why mechanistic conclusions should be drawn cautiously unless supported by convergent evidence from structural characterization, deposition studies, and barrier-relevant functional assays.

The final dosage form also affects the deposition–permeation balance. Transferosomes are frequently incorporated into gels, hydrogels, or mucoadhesive systems rather than administered as simple dispersions. These secondary matrices can alter vesicle mobility, local concentration at the surface, hydration behavior, and drug release kinetics, thereby influencing whether the formulation favors local retention or deeper permeation [33,61]. In practice, this means that a “transferosomal effect” is often inseparable from the pharmaceutical vehicle in which the vesicles are delivered. A formulation intended for superficial dermatological therapy may deliberately exploit this by combining transferosomal retention with slower release, whereas a system designed for transdermal transport may aim for a different balance.

Taken together, the available evidence suggests that transferosome-mediated delivery should not be reduced to a binary choice between intact vesicle penetration and prior drug release. Both processes may contribute, and their relative importance depends on drug physicochemical properties, vesicle composition, barrier structure, surrounding medium, and dosage-form design [14,15,16,25,33,61,77,78]. The most informative interpretation is therefore mechanistic but plural: transferosomes can enhance tissue deposition and permeation because they establish a favorable interface with the barrier, retain and release the drug in a formulation-dependent manner, and, in at least some cases, may preserve vesicular organization long enough for partial vesicle-mediated transport to matter. From a pharmaceutical standpoint, the key objective is not to prove one universal mechanism, but to identify which combination of vesicle integrity, release behavior, and barrier interaction generates the desired deposition or permeation profile for a given therapeutic purpose.

## 6. Therapeutic Applications and Translational Perspective

After considering transferosome design, characterization, deformability, and interaction with biological barriers, the next step is to evaluate their therapeutic relevance. From a pharmaceutical perspective, transferosomes are not important simply because they are ultradeformable vesicles, but because that structural and functional profile may translate into practical benefits in drug delivery. These benefits include improved local deposition, enhanced trans-barrier transport, better bioavailability of poorly permeable compounds, and the possibility of non-invasive delivery for drugs that are otherwise difficult to administer effectively [14,17,19,37]. At the same time, the therapeutic value of transferosomes cannot be judged from formulation properties alone; it must be assessed in relation to the disease target, the route of administration, the nature of the drug, and the extent to which the system offers a real advantage over other vesicular or non-vesicular approaches [14,17,19]. This section, therefore, examines the major therapeutic applications of transferosomes and places them within a broader translational context.

### 6.1. Major Therapeutic Applications of Transferosomes

The therapeutic applications of transferosomes have expanded considerably beyond their original positioning as transdermal carriers for small molecules. Although dermal and transdermal delivery still represent the most established application domain, the recent literature shows that transferosomes are now being explored in a broader range of therapeutic settings, including anti-inflammatory therapy, antimicrobial delivery, skin cancer treatment, ocular delivery, mucosal administration, and selected brain-targeting strategies via the intranasal route [14,17,19,37]. This diversification reflects the flexibility of the platform: transferosomes can accommodate hydrophilic and lipophilic drugs, can be integrated into gels and other semisolid dosage forms, and can be tailored for local, regional, or systemic delivery depending on the barrier being targeted [17,19,37].

The largest and most mature body of work remains in dermal and transdermal delivery. This is not surprising, since transferosomes were originally developed to address the limitations imposed by the stratum corneum and to improve the topical or transdermal administration of compounds with poor passive skin permeation [19,37]. Recent reviews continue to highlight transdermal drug delivery as the dominant application area, encompassing analgesics, corticosteroids, antioxidants, antihypertensives, anti-arthritic agents, and phytochemicals [17,19]. Within this category, transferosomes have often been proposed as a means of increasing drug penetration while reducing systemic exposure and improving patient compliance relative to oral or parenteral therapy [17,19].

One important therapeutic cluster is anti-inflammatory and anti-arthritic delivery. In these applications, transferosomes are particularly attractive because they may enhance local deposition in inflamed tissues while avoiding gastrointestinal and systemic adverse effects associated with oral treatment. For example, asiatic acid-loaded transferosomal gels were shown to improve skin permeation and anti-inflammatory performance, supporting the use of transferosomes for localized anti-inflammatory therapy [33]. Likewise, optimization studies on transferosomal systems co-loaded with teriflunomide and quercetin have been proposed for rheumatoid arthritis treatment, illustrating how transferosomes can be adapted for more complex anti-inflammatory strategies involving multiple actives [22]. These examples suggest that the therapeutic relevance of transferosomes in inflammatory disorders lies not only in improved permeation, but also in their ability to sustain drug residence at the site of action.

Another major application area is antimicrobial and antifungal delivery. Transferosomal systems have been explored to improve the topical treatment of infections by enhancing drug penetration into affected tissues and sustaining local drug levels. A recent example is the development of fluconazole-embedded transferosomal gels, which were optimized for antifungal activity and compatibility and were proposed as improved topical systems relative to conventional formulations [32]. Earlier work also used transferosomal approaches for localized antimicrobial delivery in dermal settings, reinforcing the view that vesicle-mediated enhancement of tissue exposure can be particularly valuable in infections where local drug concentration is a major determinant of efficacy [17,19,32].

Transferosomes have also attracted considerable attention in skin cancer and dermatological oncology. This field has been of special interest because many anticancer agents used in cutaneous malignancies require improved local delivery and deeper penetration into diseased skin while minimizing systemic toxicity. Rai et al. reviewed the state of the art of transferosomes in skin cancer therapy and highlighted their potential in conditions such as actinic keratosis, basal cell carcinoma, squamous cell carcinoma, melanoma, and Kaposi’s sarcoma [14]. More recent experimental work has continued this trend. Genistein transferosome-embedded topical systems have been investigated for melanoma treatment, while transferosomal platforms have also been proposed for antioxidant and anti-aging applications involving oxidative stress-related skin damage [46,61]. In these contexts, transferosomes are valued not only as penetration enhancers but as carriers capable of creating a more favorable drug reservoir within the skin.

A newer but rapidly growing application area is ocular delivery. Ocular barriers severely restrict topical drug bioavailability, and transferosomes have emerged as promising carriers because they may improve surface adaptation, tissue penetration, and retention without requiring invasive administration [21,51]. Curcumin-loaded transferosomes have shown enhanced penetration into multiple ocular tissues after topical application, illustrating the capacity of the platform to improve the delivery of poorly soluble bioactives in ophthalmic settings [21]. Tonabersat transferosomes have likewise been developed for ocular delivery, supporting the broader idea that transferosomes can be adapted beyond skin applications when vesicle deformability is aligned with the specific constraints of the ocular surface [51].

Mucosal and intranasal applications also represent an increasingly relevant domain. Here, the therapeutic interest lies in improving residence and absorption at hydrated, clearance-prone surfaces, and, in some cases, enabling delivery to the central nervous system through the nose-to-brain pathway [81,82]. Intranasal transferosomal systems containing resveratrol and donepezil have been developed to enhance bioavailability and support brain-targeted delivery, demonstrating that the transferosome concept may also be useful outside classical dermal delivery when combined with appropriate retention-promoting formulation strategies [81,82].

Taken together, these application areas show that transferosomes have evolved from a mainly transdermal delivery concept into a broader therapeutic platform. Even so, the field remains unevenly developed. Dermal and transdermal use is still the most strongly established and best supported domain, whereas ocular and mucosal applications, although promising, are more recent and require further mechanistic and translational validation [17,19,21,37,51,81,82]. This unevenness is important for interpretation: it suggests that the therapeutic promise of transferosomes is real, but also that their strongest evidence base still lies in applications where barrier adaptation, local retention, and non-invasive delivery are directly aligned with the intrinsic strengths of the platform.

To further clarify the practical significance of these applications, representative transferosome formulations and their remaining unmet needs are summarized in Table 4. The aim of this comparison is not to provide an exhaustive catalog of all reported formulations, but to highlight how reported advantages remain linked to formulation-specific limitations, including incomplete mechanistic validation, limited interstudy comparability, insufficient long-term stability data, scalability concerns, and scarce clinical evidence.

Overall, these examples show that transferosomes have generated promising results across several routes of administration, but they also reveal a recurrent gap between formulation performance and translational readiness. Most studies demonstrate improved permeation, deposition, release, or pharmacokinetic behavior, yet fewer establish which formulation attributes are mechanistically responsible for those outcomes or whether the same performance can be reproduced under standardized, scalable, and clinically relevant conditions. Therefore, the unmet need in the field is not simply the development of additional transferosome formulations, but the establishment of stronger formulation–mechanism–performance relationships supported by appropriate comparators and harmonized characterization.

### 6.2. Emerging Developments in Transferosome-Based Delivery

Recent transferosome research is moving beyond classical dermal and transdermal delivery toward more specialized systems in which vesicle deformability is combined with additional formulation or functional strategies. One important trend is the development of hybrid transferosome-based platforms, including transferosomal gels, hydrogels, microneedle-assisted systems, and vesicular systems positioned in relation to ethosomes and transethosomes [15,32,33,45,55,60,85]. These approaches seek to combine the membrane adaptability of transferosomes with improved residence time, mechanical assistance, local retention, or complementary penetration-enhancement mechanisms. However, hybridization also increases formulation complexity and may make it more difficult to identify whether improved performance arises from vesicle deformability, the secondary dosage form, the added enhancement strategy, or the interaction among all these elements [15,55,86].

Targeted and surface-modified transferosomes are another emerging direction, particularly for routes in which residence time and biological-interface recognition are critical. For example, hyaluronic-acid-doped nanotransfersomes have been investigated for intranasal donepezil delivery, illustrating how surface modification can be used to combine deformable vesicles with mucoadhesive or targeting-oriented functionality [82]. More broadly, intranasal transferosome-based systems containing resveratrol, donepezil, and carvedilol show that nose-to-brain and mucosal applications increasingly require integration of vesicle deformability with retention-promoting formulation design [81,82,83]. These examples suggest that future targeted transferosome systems should be evaluated not only for drug loading and permeation, but also for residence time, interaction with local fluids, biological selectivity, and mechanistic evidence supporting the claimed targeting effect [81,82,83].

Ocular delivery has also become a relevant emerging application. Curcumin-loaded transferosomes and tonabersat transferosomes have shown that deformable vesicles can be adapted for topical ocular delivery, where tear turnover, blinking, nasolacrimal drainage, and tissue-specific penetration strongly influence performance [21,49,51]. Importantly, studies showing that tear-fluid properties can modify transferosome size, polydispersity, zeta potential, mucoadhesion, and ocular penetration behavior indicate that ocular transferosomes should be evaluated under application-relevant conditions rather than only in purified aqueous media [49]. This reinforces the broader principle that emerging applications require barrier-specific characterization instead of direct extrapolation from skin-delivery models.

Pulmonary delivery represents a less consolidated but scientifically relevant direction. Transferosome studies involving pulmonary administration highlight that phospholipid, surfactant, and cholesterol selection can influence vesicle performance when formulations are adapted to nebulization or respiratory delivery conditions [26]. In this context, the main formulation challenge is different from that encountered in skin or ocular delivery: vesicles must tolerate aerosolization-related stress, maintain acceptable size and stability, and interact appropriately with the respiratory interface. Therefore, pulmonary transferosome development requires attention not only to deformability, but also to aerosol performance, post-nebulization vesicle integrity, drug retention, and safety of inhaled excipients [26].

Gene and nucleic-acid delivery remains more exploratory for transferosome-based systems than for other lipid nanocarriers. Nevertheless, the broader drug-delivery literature shows that nucleic-acid therapeutics are strongly limited by biological barriers, tissue specificity, intracellular access, and structural requirements of lipid-based carriers [2,3,53]. Transferosomes could, in principle, contribute to this area if their deformable bilayer architecture can be adapted to protect labile macromolecules and support interaction with non-invasive barriers. However, this direction should be regarded as prospective rather than established, because convincing transferosome-specific evidence would require demonstration of nucleic acid stability, encapsulation or association efficiency, protection from degradation, cellular uptake, biological activity, and safety under relevant delivery conditions [2,3,53].

Stimuli-responsive and AI-assisted transferosome design are also promising future directions, but they remain less mature in the transferosome-specific literature than QbD-based optimization. Stimuli-responsive systems could be useful if external or local triggers are used to regulate drug release, vesicle restructuring, or barrier interaction, but such approaches must demonstrate that the stimulus provides a reproducible functional advantage rather than merely adding complexity. Similarly, AI-assisted formulation design may become valuable for identifying relationships among composition, process variables, CQAs, deformability, and biological outcomes, especially as larger and more standardized datasets become available. At present, however, QbD and design-of-experiments approaches provide the clearest development-oriented framework already visible in transferosome research [12,20,22,72,73].

Microfluidic and manufacturing-aware approaches are likely to become increasingly important for the translation of transferosomes. Current literature still relies heavily on thin-film hydration, sonication, extrusion, and other laboratory-scale methods, which are useful for exploratory studies but can be difficult to standardize during scale-up [12,17,19,37]. Recent discussions on lipid nanocarriers and QbD-based product development emphasize the need for scalable unit operations, robust design spaces, and stronger links between critical material attributes, critical process parameters, and final product performance [12,37,72,73]. For transferosomes, microfluidic or continuous-manufacturing strategies may therefore offer future advantages by improving control over vesicle size, population uniformity, reproducibility, and batch-to-batch comparability [12,37,72,73].

Overall, these emerging developments indicate that the next phase of transferosome research will likely be defined less by the simple demonstration of deformability and more by the integration of deformability with targeting, hybrid dosage forms, route-specific characterization, scalable manufacturing, and data-driven formulation design. The field should therefore move toward application-specific transferosome platforms in which composition, processing, CQAs, mechanism, safety, and translational feasibility are evaluated together.

### 6.3. Safety, Toxicity, and Biocompatibility Considerations

Safety evaluation is an essential requirement for transferosome development because these systems are intended to interact closely with biological barriers and, in many cases, to remain at the site of administration for prolonged periods. Although transferosomes are generally based on phospholipids and pharmaceutically familiar excipients, their safety cannot be inferred from composition alone. The inclusion of edge activators, charge modifiers, cholesterol, secondary vehicles, and high drug loads may alter membrane interaction, local tolerability, cellular response, and long-term biocompatibility [12,19,25,26,27]. Therefore, transferosomes should be evaluated not only as drug carriers, but also as biologically active interfaces whose safety depends on the complete formulation.

Cytotoxicity is one of the first safety endpoints that should be addressed. In vitro cell-viability assays are commonly used to evaluate whether the vesicular formulation, empty carrier, drug-loaded system, or final gel vehicle compromises cellular metabolic activity or membrane integrity [32,46,51,60]. This distinction is important because toxicity may arise from the drug, the surfactant, the lipid composition, the charge modifier, the secondary dosage form, or their combined interaction. Recent transferosome-based topical, ocular, and hydrogel formulations have incorporated compatibility or cytotoxicity-related evaluations as part of the development process, reflecting the need to connect formulation performance with local biological tolerance [32,46,51,60].

For dermal and transdermal applications, skin irritation and local tolerability are particularly important. Edge activators are required to generate membrane deformability, but they may also perturb biological lipid domains, modify barrier organization, or increase local irritation if used at excessive concentrations [12,19,23,25]. This creates a formulation trade-off: the same component that improves membrane softness and barrier interaction may also increase the risk of leakage, barrier disruption, or local intolerance when the phospholipid-to-edge-activator ratio is not properly optimized [12,23,25]. Consequently, safety evaluation should include not only drug-loaded formulations, but also blank transferosomes and surfactant-containing controls, so that the contribution of the vesicular carrier can be distinguished from that of the active compound.

Surface charge and auxiliary excipients also influence biological safety. Cationic or charge-modified transferosomes may improve colloidal stability, drug association, or interaction with negatively charged biological surfaces, but they may also increase membrane interaction and potential cytotoxicity depending on charge density and excipient type [19,27]. Cholesterol can improve bilayer cohesion and reduce excessive leakage, but it may also alter deformability and release behavior, indirectly affecting local exposure to both drug and excipients [26,27]. These examples reinforce that auxiliary components should be considered safety-relevant formulation variables rather than secondary excipients.

Long-term biocompatibility remains less developed in the transferosome literature than short-term physicochemical and permeation testing. This gap is important because transferosomal formulations may be used repeatedly, especially in chronic dermatological, ocular, inflammatory, or mucosal conditions. Long-term safety evaluation should therefore consider repeated-dose tolerability, persistence of excipient exposure, possible barrier remodeling, inflammatory response, and the stability of the formulation during storage and use [17,47,57,58,59]. Physical or chemical degradation during storage may also have safety implications, since lipid oxidation, hydrolysis, vesicle aggregation, or drug leakage can modify both product quality and biological response [57,58,59].

Immunogenicity is another relevant but insufficiently explored issue. Conventional phospholipid-based vesicles are often regarded as biocompatible, but transferosomes may include surfactants, bile salts, charged excipients, or surface-modifying components that influence recognition by epithelial, mucosal, ocular, or immune-associated cells [12,19,25,27]. For most topical or mucosal transferosome systems, the immediate concern is local inflammation or irritation rather than systemic immunogenicity. However, as transferosomes are increasingly adapted for ocular, intranasal, pulmonary, and macromolecular delivery, immunological compatibility should become a more explicit part of formulation assessment [21,26,49,51,81,82,83].

A rigorous safety strategy should therefore distinguish between carrier-related safety, drug-related safety, and product-related safety. Carrier-related safety concerns the phospholipid, edge activator, charge modifier, cholesterol, and stabilizing excipients. Drug-related safety concerns how vesicular incorporation modifies local exposure, retention, release, and tissue distribution. Product-related safety concerns the final dosage form, storage stability, route of administration, repeated-use conditions, and manufacturing consistency [17,64,70]. This distinction is particularly relevant for regulatory translation because a transferosomal formulation cannot be considered safe simply because its individual components are known excipients; safety must be demonstrated for the complete vesicle–drug–vehicle system under route-relevant conditions [64,70].

Overall, safety and biocompatibility should be integrated early into transferosome design rather than evaluated only after permeation or efficacy has been optimized. Future studies should more consistently report cytotoxicity, local irritation, blank-carrier controls, repeated-exposure data, formulation stability under use conditions, and route-specific tolerability. This would strengthen the translational credibility of transferosome-based products and help determine whether improved delivery performance is achieved without compromising biological safety.

### 6.4. Patents, Intellectual Property, and Commercialization Prospects

The industrial relevance of transferosomes depends not only on their biological performance, but also on whether the technology can be protected, manufactured, regulated, and differentiated in a commercially meaningful way. From an intellectual-property perspective, transferosome-related inventions may involve several protectable elements, including vesicle composition, phospholipid-to-edge-activator ratio, drug-specific formulations, preparation methods, stabilization strategies, final dosage forms, and route-specific therapeutic applications. This is particularly relevant because transferosomes are not defined by a single material, but by a formulation logic in which membrane deformability, drug retention, barrier interaction, and product performance are generated through the combined effect of composition and process variables [12,17,37].

A major challenge for intellectual-property positioning is that many foundational aspects of transferosome technology are already well established. As a result, broad claims directed simply to phospholipid vesicles containing surfactants may be difficult to defend unless they are linked to a clearly differentiated formulation, therapeutic use, manufacturing method, stability profile, or performance advantage. In practical terms, stronger patentability is more likely to arise from specific and reproducible formulation designs than from generic statements about ultradeformability. Examples include optimized drug–carrier systems, hybrid transferosomal gels, ocular or intranasal transferosome formulations, lyophilized or stabilized products, and manufacturing approaches that provide improved control over vesicle size, deformability, storage stability, or batch reproducibility [17,21,31,37,51,72,73].

Current intellectual-property trends in advanced vesicular delivery increasingly favor development-oriented claims. These include claims based on critical material attributes, critical process parameters, defined quality profiles, improved stability, route-specific performance, and scalable manufacturing strategies. This trend is consistent with the broader movement toward QbD-based formulation development and manufacturing-aware lipid nanocarrier design [12,20,22,72,73]. For transferosomes, this means that future IP value will likely depend on demonstrating that a given formulation is not only deformable, but also reproducible, stable, scalable, safe, and superior to relevant comparators for a defined therapeutic purpose [12,17,37,64,72,73].

Commercialization prospects are strongest in application areas where transferosomes offer a clear practical advantage over conventional dosage forms or competing nanocarriers. Dermal and transdermal delivery remain the most mature opportunities because they align closely with the original strengths of the platform: local deposition, non-invasive administration, and improved interaction with the stratum corneum [17,19,37]. Ocular, intranasal, buccal, and mucosal applications may also offer attractive development opportunities, but these areas require stronger route-specific validation, safety assessment, and demonstration of clinically relevant performance advantages [21,49,51,81,82,83,84]. In each case, commercial viability will depend on whether the transferosomal product solves a real formulation or delivery problem that cannot be addressed more simply by conventional liposomes, emulsions, gels, or other vesicular systems [15,17,19].

Several barriers still limit commercialization. These include insufficient interstudy comparability, lack of standardized deformability testing, limited long-term stability data, uncertain scale-up routes, incomplete safety packages, and the absence of a transferosome-specific regulatory pathway [17,37,64,72,73]. These issues directly affect industrial decision-making because a formulation with promising permeation data may still be commercially weak if it cannot be manufactured reproducibly, stored under realistic conditions, protected through defensible IP, or justified against simpler delivery technologies. Therefore, commercialization of transferosomal systems will require a more integrated development strategy in which formulation performance, patentability, manufacturability, regulatory expectations, safety, and market differentiation are considered together [64,72,73].

Overall, the patent and commercialization landscape suggests that transferosomes should be advanced as product-specific pharmaceutical technologies rather than as a generic nanocarrier category. The most commercially credible transferosome products will likely be those that combine a defensible formulation design, a clear therapeutic need, reproducible quality attributes, acceptable safety, scalable manufacturing, and a demonstrable advantage over existing delivery options. This reinforces the broader conclusion of the present review: the future value of transferosomes will depend less on the novelty of the term itself and more on the ability to convert deformable vesicle science into robust, differentiated, and clinically useful pharmaceutical products.

### 6.5. Comparative Positioning Versus Other Vesicular Systems

The pharmaceutical relevance of transferosomes becomes clearer when they are positioned against other vesicular systems used for barrier-oriented drug delivery, especially conventional liposomes, ethosomes, and transethosomes [15,17,19]. These carriers share the broad objective of improving drug transport across biological interfaces, but they differ in membrane composition, dominant penetration mechanism, colloidal behavior, and practical formulation implications [15,17,19]. For this reason, the choice among them should not be based on nomenclature or popularity, but on the relationship between vesicle architecture, drug properties, target barrier, and intended therapeutic outcome [17,19]. The main comparative features of transferosomes and related vesicular systems are summarized in Table 5.

Conventional liposomes represent the most basic comparator. They are typically composed of phospholipid bilayers with or without cholesterol and are valued for their biocompatibility, drug-encapsulation versatility, and long-standing pharmaceutical relevance [78]. However, in topical and transdermal settings, conventional liposomes generally show limited penetration across intact skin because their bilayers are more rigid and they often remain confined to superficial layers or act mainly as local reservoirs [19,78]. In this context, transferosomes are usually considered advantageous when enhanced barrier adaptation is needed, since the inclusion of edge activators gives them greater capacity to respond to mechanical confinement than conventional liposomes [17,19]. This distinction does not imply that transferosomes are universally superior; rather, it means that they may be preferable when local or trans-barrier delivery depends strongly on vesicle deformability and interfacial adaptability [17,19,78].

Ethosomes differ from transferosomes in that their defining compositional feature is a relatively high ethanol content, which contributes both to vesicle softness and to perturbation of barrier lipids [15,85]. In practical terms, ethosomes often rely more strongly on ethanol-mediated enhancement of skin penetration, whereas transferosomes rely more explicitly on membrane deformability induced by edge activators [15,19,85]. Comparative studies have shown that both systems can improve dermal delivery relative to conventional liposomes, but their relative performance depends on the drug and formulation context. In the comparative work of Ascenso et al., transfersomes, ethosomes, and transethosomes all showed formulation-dependent differences in deformability, drug loading, and skin-delivery behavior, supporting the view that none of these platforms can be ranked universally without regard to composition and payload [15]. Likewise, comparative studies with specific actives have shown that ethosomes may outperform transferosomes in some cases, whereas transferosomes may be more effective in others, depending on how ethanol content, surfactant composition, and drug affinity shape the final vesicle–barrier interaction [87].

Transethosomes occupy a more hybrid position because they combine phospholipids, ethanol, and an edge activator, thereby integrating features associated with both ethosomes and transferosomes [15,86]. For this reason, they are often described as more compositionally complex and potentially more flexible systems, particularly in transdermal delivery [15,86]. Comparative literature suggests that transethosomes may sometimes achieve stronger permeation than either ethosomes or transferosomes, likely because they couple ethanol-mediated barrier modulation with surfactant-assisted membrane adaptability [15,86]. However, this apparent advantage comes with formulation complexity: transethosomes may also present additional challenges related to stability, component optimization, and mechanistic interpretation, since it becomes harder to distinguish the contribution of ethanol from that of the edge activator [15,86]. In this sense, transferosomes may remain preferable when a simpler and more mechanistically focused ultradeformable system is desired, whereas transethosomes may be attractive when maximizing penetration is prioritized over compositional simplicity [15,86].

A useful way to position these systems is therefore by their dominant design logic. Conventional liposomes prioritize biocompatible vesicular encapsulation but are comparatively limited in barrier negotiation. Transferosomes prioritize mechanical adaptability through edge-activator-mediated membrane softening. Ethosomes prioritize ethanol-driven interaction with barrier lipids and improved partitioning into the barrier. Transethosomes combine ethanol and edge activators in an attempt to amplify both mechanisms [15,19,85,86]. This comparative framework is more informative than asking which vesicle type is “best,” because the answer depends on whether the formulation goal is superficial deposition, deeper tissue permeation, sustained local release, or enhanced systemic absorption after non-invasive administration [15,17,19].

The comparative positioning also depends on the nature of the drug. Highly lipophilic compounds may benefit strongly from ethosomal or transethosomal systems if ethanol improves solubilization and barrier partitioning, while drugs requiring stronger vesicle-associated transport or more controlled membrane-mediated adaptation may be better suited to transferosomes [19,85,87]. For sensitive payloads, including biologically active molecules or drugs whose retention in the vesicle matters for delivery performance, the simpler architecture of transferosomes may offer advantages over ethanol-rich systems that can impose different stability constraints [17,19]. This does not establish a rigid hierarchy, but it does suggest that transferosomes are especially well positioned when the formulation objective depends on balancing deformability, drug retention, and controlled interaction with the barrier.

From a translational point of view, transferosomes occupy an intermediate position between simplicity and performance enhancement. They are more functionally specialized than conventional liposomes, but usually less compositionally complex than transethosomes [15,17,19,86]. This may be advantageous when formulation development seeks a platform with strong barrier-adaptive properties without introducing excessive formulation complexity. At the same time, the comparative literature makes clear that transferosomes should not be treated as a default superior system. Their relevance lies in offering a distinct and useful formulation logic, not in replacing all other vesicular carriers across all applications [15,17,19].

Lipid nanoparticles represent a related but distinct comparator because their dominant design logic is not vesicle deformability, but the formation of lipid-based nanostructures optimized for payload protection, systemic delivery, and, in many cases, nucleic-acid transport [2,53,57]. Compared with transferosomes, lipid nanoparticles generally offer stronger relevance for parenteral or systemic delivery of labile macromolecules, while transferosomes are more closely aligned with non-invasive or minimally invasive barrier-oriented delivery. This distinction is important because both systems belong to the broader lipid nanocarrier field, but they are usually optimized for different therapeutic routes, payload classes, and performance criteria. Therefore, transferosomes should not be evaluated as direct substitutes for lipid nanoparticles; rather, they should be positioned as deformable vesicular systems whose main value lies in local, topical, transdermal, ocular, mucosal, or intranasal applications where membrane adaptability and interfacial behavior are central to the delivery problem [2,17,19,53,57].

For this reason, transferosomes are best viewed as one member of a broader family of barrier-oriented vesicular systems, with a particular strength in applications where deformability, interfacial adaptation, and non-invasive delivery are tightly linked to therapeutic success [15,17,19]. Their comparative value is greatest when the formulation question is framed properly: not whether transferosomes are better in general, but whether they offer the most coherent balance of composition, mechanics, and performance for the delivery problem at hand.

### 6.6. Translational Barriers, Regulatory Challenges, Manufacturing Scale-Up, Market Potential, and Future Directions

Despite the extensive experimental interest in transferosomes, their transition from promising laboratory systems to robust pharmaceutical products remains limited [17,64]. This gap is not necessarily due to a lack of biological potential, but rather to the fact that transferosomal performance depends on multiple interdependent variables that are difficult to control simultaneously at the level required for clinical development, industrial manufacturing, and regulatory evaluation [17,72]. In the recent literature, the main barriers to translation are consistently linked to reproducibility, analytical comparability, manufacturability, storage stability, safety evaluation, regulatory uncertainty, and the absence of sufficiently standardized development frameworks [17,37,64,72,73].

From a regulatory perspective, transferosomes occupy a complex position because they are neither simple conventional liposomes nor a formally distinct regulatory category. In practice, they would likely be evaluated within broader frameworks applicable to lipid-based nanocarriers, liposomal products, or complex drug-delivery systems, where regulators expect robust characterization, process control, batch comparability, safety assessment, and a clear justification of critical quality attributes [64]. This has a direct implication for clinical translation: claims regarding deformability, improved barrier penetration, or superiority over other vesicular systems must be supported by standardized and development-relevant datasets rather than by isolated exploratory studies [17,64].

A second barrier is batch reproducibility. Transferosomes are highly sensitive not only to nominal composition, but also to process variables such as solvent removal, hydration conditions, energy input, extrusion or sonication regime, and post-processing history [17,72,73]. Small changes in those variables can alter vesicle size distribution, drug association, deformability, and stability, thereby making it difficult to reproduce a given formulation reliably from batch to batch [17,72]. This problem is well aligned with the broader Quality by Design literature, which emphasizes that robust product development requires early identification of critical material attributes and critical process parameters rather than late empirical adjustment [72,73].

Scale-up and large-scale manufacturability remain equally important constraints. A large part of the transferosome literature still relies on laboratory-scale methods such as thin-film hydration followed by sonication or extrusion. These approaches are useful for exploratory formulation work, but they are not always well-suited to industrial translation because they can be batch-dependent, labor-intensive, difficult to standardize, and sensitive to solvent removal, hydration conditions, and post-processing history [17,73]. This concern is especially relevant because the current review shows that transferosome performance is strongly influenced by preparation method, vesicle size distribution, lamellarity, deformability, drug retention, and storage stability [12,17,19,37]. Recent discussions on lipid nanocarriers have therefore stressed the need to move toward scalable unit operations, manufacturing-aware design spaces, and QbD-guided control strategies capable of linking critical material attributes and critical process parameters with final product performance [72,73]. For transferosomes, this means that promising bench-scale performance is not enough; the same vesicle quality must also be reproducible under scale-up conditions, compatible with realistic manufacturing workflows, and stable over commercially relevant storage periods [12,37,72,73].

Another major issue is physical and chemical stability. Transferosomes are intentionally designed as soft vesicular systems, which makes them attractive for barrier-oriented delivery but also potentially more vulnerable to aggregation, leakage, hydrolysis, oxidation, and structural rearrangement during storage [17,37]. From a translational perspective, short-term characterization immediately after preparation is insufficient. A viable product must preserve drug content, vesicle integrity, and functionally relevant performance over realistic storage periods and conditions [17,37,64]. This is one reason why future transferosome development will need to place greater emphasis on stabilization strategies, including rational excipient selection, drying approaches, and dosage-form integration.

A further barrier is regulatory uncertainty. There is no transferosome-specific regulatory framework, so these systems would most likely be evaluated within broader pathways applicable to lipid-based nanocarriers or liposomal products [64]. Under such a framework, strong emphasis would be placed on analytical characterization, process control, batch comparability, and justification of critical quality attributes [64,72]. This has an important implication for transferosome research: claims about deformability, barrier penetration, or superiority over other vesicular systems will need to be supported by more standardized and development-relevant datasets than are typically provided in exploratory academic studies [17,64,72].

There is also a conceptual barrier linked to the mechanism. Transferosomes are often described through persuasive mechanistic narratives, particularly around deformability and barrier transport, but these narratives are not always matched by equally rigorous and harmonized experimental evidence [17,37]. From a translational standpoint, this matters because formulation development benefits from mechanisms that are sufficiently clear to guide comparability criteria, performance specifications, and formulation decisions. If the field continues to rely on broad explanatory labels while using heterogeneous measurement methods, translation will remain slowed by uncertainty over which properties must actually be controlled and validated [17,37,64].

The most productive future direction is therefore not simply to make transferosomes more elaborate, but to make them more predictable [17,72,73]. This includes wider adoption of Quality by Design principles, better harmonization of deformability and release testing, stronger orthogonal characterization packages, and more explicit links between formulation attributes and therapeutic objectives [17,72,73]. It also implies a shift from asking whether transferosomes “work” in general to asking which transferosomal design is appropriate for a defined drug class, barrier type, and dosage form [17,37,73].

A second important direction is application-focused specialization. Transferosomes are unlikely to become universally preferred vesicular systems across every indication. Their strongest future may lie in therapeutic contexts where membrane adaptability, local retention, and non-invasive delivery offer a clear functional advantage, such as selected dermatological, ocular, and mucosal applications [17,37]. In those areas, the most influential studies will likely be those that combine rigorous physicochemical characterization, clinically relevant barrier models, scalable manufacturing logic, and a transparent rationale for why the transferosomal platform is preferable to competing systems in that specific setting [17,37,64,72,73].

The market potential of transferosomes should therefore be understood as application-specific rather than universal. The most credible opportunities are expected in areas where the platform addresses a clear unmet delivery need, particularly dermal and transdermal therapy, ocular delivery, selected mucosal applications, and intranasal or nose-to-brain strategies [17,21,37,49,51,81,82,83,84]. However, commercial value will depend on demonstrating a practical advantage over simpler or more established technologies, including conventional liposomes, gels, emulsions, ethosomes, transethosomes, or other lipid-based nanocarriers [15,17,19]. In this sense, the strongest future candidates will not necessarily be the most complex transferosome formulations, but those that combine a clear therapeutic rationale, defensible product differentiation, reproducible manufacturing, acceptable safety, regulatory plausibility, and clinically meaningful performance [64,72,73].

Taken together, these considerations indicate that future transferosome research should move beyond descriptive formulation reports toward more hypothesis-driven and decision-oriented studies. Priority should be given to formulations that define a clear therapeutic problem, justify the selection of transferosomes over simpler delivery systems, identify the CQAs most relevant to that specific application, and link those attributes to mechanism, safety, stability, manufacturability, and clinically meaningful performance. Such a framework would help distinguish genuinely promising transferosomal products from formulations that are interesting at the laboratory level but insufficiently differentiated for pharmaceutical development.

**Figure 3:**
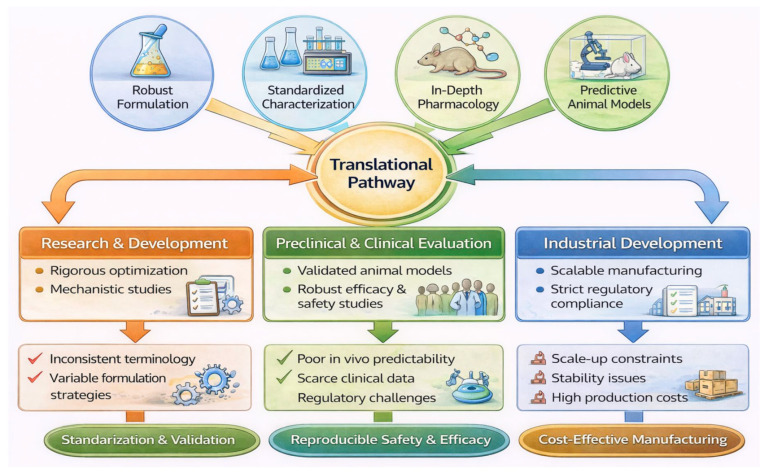
Schematic overview of the key stages involved in the development and clinical translation of transferosome formulations, including research and development, preclinical and clinical evaluation, and industrial manufacturing. The figure highlights critical bottlenecks at each stage, such as inconsistent terminology and formulation variability, limited in vivo predictability and scarce clinical data, as well as scale-up constraints and regulatory challenges. Addressing these limitations is essential to achieve standardized, reproducible, and cost-effective transferosome-based therapeutics.

A schematic overview of the main translational pathway and associated bottlenecks for transferosome-based drug delivery systems is presented in Figure 3.

## 7. Conclusions

Transferosomes have progressively evolved from a formulation concept closely associated with ultradeformable transdermal vesicles into a broader drug-delivery platform with relevance across several non-invasive and minimally invasive administration routes. Their enduring scientific interest lies in a distinctive formulation logic: they do not merely encapsulate drugs within a phospholipid vesicle, but intentionally engineer the membrane itself as a dynamic interface capable of adapting to biological constraints. In that sense, transferosomes occupy a singular position within the broader family of vesicular carriers, not because they are universally superior, but because they place membrane adaptability at the center of delivery design.

One of the clearest conclusions emerging from the available literature is that transferosomes should not be interpreted as simple liposomes containing surfactants. Their behavior is not defined by composition alone, but by the integrated relationship among phospholipid organization, edge activator content, auxiliary excipients, drug physicochemical properties, preparation method, vesicle architecture, and the conditions under which the system is evaluated. This is precisely why transferosome performance is often highly formulation-specific. Small changes in membrane composition or process history may alter not only vesicle size, drug loading, and stability, but also deformability, release kinetics, and barrier interaction in ways that are therapeutically meaningful.

Deformability remains the conceptual hallmark of transferosomes and, at the same time, one of the most difficult properties to interpret rigorously. The literature reviewed here strongly supports its importance, but it also shows that deformability is too often invoked as a broad explanatory label rather than handled as a method-dependent functional property. It is not synonymous with membrane fluidity, nor is it a universally predictive marker of biological performance. A highly deformable vesicle is not necessarily a more effective delivery system unless that deformability is accompanied by sufficient structural integrity, adequate drug retention, appropriate release behavior, and productive interaction with the target barrier. The scientific challenge, therefore, is not to reaffirm deformability as a defining slogan, but to understand the specific conditions under which it becomes pharmaceutically advantageous.

A related conclusion concerns the mechanism of transferosome-mediated delivery. The evidence does not support a rigid, one-dimensional view in which transferosomes either penetrate biological barriers intact or fail to do so. Rather, the literature points toward a more layered interpretation in which intact vesicle transport, partial penetration, local vesicle restructuring, interfacial lipid exchange, barrier modulation by surfactants, and drug release at or within the barrier may all contribute to the final therapeutic outcome. The relative importance of each process appears to depend on the drug, the barrier, the formulation, and the dosage form. This mechanistic plurality should not be seen as a weakness of the field, but it does demand greater discipline in how conclusions are drawn. Transferosomal systems should be judged not by their conformity to a single mechanistic narrative, but by the coherence between their physicochemical behavior and their biological performance.

From an application standpoint, the strongest and most mature evidence still lies in dermal and transdermal delivery, where transferosomes have repeatedly shown value as systems capable of improving local deposition, enhancing permeation of difficult actives, and supporting non-invasive therapeutic strategies. More recent work in ocular and mucosal delivery extends this promise into other hydrated and clearance-sensitive biological environments, suggesting that the utility of transferosomes is not confined to the skin. Even so, these newer application domains remain less consolidated than the transdermal field and will require more robust mechanistic, comparative, and translational validation before their full relevance can be established. What emerges from the current evidence is not a universal platform equally optimized for all uses, but a versatile formulation approach whose strengths become most evident when barrier adaptation and local interfacial control are central to therapeutic success.

The review also makes clear that the main limitations of the field are no longer conceptual novelty, but methodological inconsistency and translational fragility. Transferosome research has produced an extensive body of promising experimental work, yet this progress has not been matched by equivalent harmonization in characterization methods, deformability testing, reporting quality, or formulation benchmarking. The consequence is literature rich in positive claims but comparatively poor in reproducibility and direct comparability. This is especially problematic at a stage where the field increasingly seeks pharmaceutical relevance rather than proof-of-concept novelty. If transferosomes are to advance toward realistic product development, the emphasis must shift from demonstrating isolated formulation success to establishing robust analytical frameworks capable of supporting reproducible quality.

In this regard, one of the most pressing priorities is the adoption of a more development-oriented mindset. Transferosomal systems will benefit from stronger integration of critical quality attributes, orthogonal characterization strategies, Quality by Design principles, and manufacturing-aware formulation logic. The future of the field is unlikely to depend on generating ever more complex vesicular variants unless those systems can also demonstrate reproducible preparation, acceptable stability, scalable processing, and a persuasive rationale for superiority over competing delivery platforms. In other words, the next important phase of transferosome research is not expansion through complexity, but consolidation through rigor.

Another important conclusion is that transferosomes should not be advanced as universally preferable alternatives to conventional liposomes, ethosomes, or transethosomes. Their comparative value is contextual. There are delivery problems for which conventional liposomes may be sufficient and more straightforward, others in which ethanol-rich systems may offer stronger barrier perturbation, and still others in which transferosomes may provide the most coherent balance between deformability, retention, and controlled barrier interaction. The critical question is therefore not whether transferosomes are “better” in a general sense, but whether their membrane mechanics and formulation behavior align more effectively with the therapeutic and biopharmaceutical demands of a specific application.

What the field now needs most is selectivity: selectivity in choosing the right applications, selectivity in defining the right mechanistic questions, and selectivity in deciding which formulation attributes truly matter for translation. The most influential future studies will likely be those that do not merely report improved permeation or higher deformability, but demonstrate how transferosome design can be rationally matched to a particular drug, barrier, and clinical purpose. Such work would move the field beyond descriptive enthusiasm and toward a more predictive, decision-oriented science of vesicular drug delivery.

Taken together, the literature supports the view that transferosomes remain one of the most compelling classes of ultradeformable vesicles in pharmaceutical nanotechnology. Their relevance is sustained not only by historical influence, but by their continued ability to frame important questions about how vesicle mechanics, drug release, and barrier biology can be brought into productive alignment. Their long-term significance, however, will depend on whether future research can combine biological ambition with stronger analytical discipline, clearer mechanistic reasoning, and a more credible translational pathway. Under those conditions, transferosomes may yet fulfill the promise that first made them distinctive: not simply as flexible vesicles, but as genuinely useful systems for advanced non-invasive drug delivery.

## Figures and Tables

**Table 1:** Main components of transferosomes and their functional roles.

Component Class	Representative Examples	Main Functional Role in the Formulation	Possible Impact on Vesicle Behavior and Performance	References
Phospholipids	Soy phosphatidyl-choline (SPC), egg phosphatidylcholine (EPC), hydrogenated phosphatidylcholine, DMPC	Form the bilayer framework; provide structural integrity; create hydrophilic and hydrophobic compartments for drug incorporation	Strongly influence vesicle formation, bilayer cohesion, permeability, fluidity, lamellarity, and encapsulation of both hydrophilic and lipophilic drugs	[17,19,26]
Edge activators	Tween 80, Span 80, sodium cholate, sodium deoxycholate, Tween 20, Span 20	Disrupt local lipid packing and increase membrane flexibility/deformability	Enhance stress adaptability and barrier interaction, but excessive amounts may increase leakage, reduce entrapment, or destabilize the vesicle	[17,19,25]
Cholesterol	Cholesterol	Modulates bilayer packing and membrane rigidity; reduces excessive permeability	May improve vesicle integrity and storage stability, but high levels can reduce deformability and modify release/permeation behavior	[26,27]
Charge-inducing agents	Stearylamine, dicetyl phosphate, cationic surfactants	Adjust surface charge and electrostatic stabilization	Affect zeta potential, aggregation tendency, interaction with ionizable drugs, colloidal stability, and in some cases tissue interaction/permeation	[27]
Aqueous phase/hydration medium	Water, phosphate buffer, saline, pH-adjusted media	Hydrates the lipid film and defines the external/internal aqueous environment	Influences drug ionization, vesicle size, zeta potential, osmotic balance, membrane hydration, retention, and stability	[17,19]
Cryo-/lyoprotectants	Sucrose, trehalose	Protect vesicles during freezing and drying; improve redispersion after reconstitution	Reduce fusion, leakage, and structural damage during lyophilization; improve long-term storage stability when properly optimized	[29,30,31]
Drug cargo	Hydrophilic, lipophilic, amphiphilic, ionizable, or macromolecular compounds	Therapeutic payload; its physicochemical nature conditions localization within the vesicle	Determines entrapment efficiency, retention, release profile, membrane perturbation, and barrier interaction; may also affect deformability and stability	[17,19,21]
Secondary vehicle/final dosage form	Gels, hydrogels, mucoadhesive systems, semisolids	Provides a practical administration platform and modulates residence time at the application site	Alters viscosity, residence time, surface contact, release behavior, and sometimes physical stability of the transferosomal dispersion	[32,33]

**Table 2:** Preparation methods and their influence on vesicle properties.

Preparation Method	Principle	Main Advantages	Main Limitations	Influence on Vesicle Properties	References
Thin-film hydration	Lipids/surfactants are dissolved in organic solvent, dried as a thin film, and then hydrated	Simple, widely used, flexible for formulation screening	Batch-dependent; sensitive to hydration conditions; usually requires post-size reduction	Good vesicle formation; size, lamellarity, and entrapment strongly depend on hydration and downstream processing	[12,19,38,39]
Ethanol injection/solvent displacement	The lipid phase in ethanol is injected into the aqueous phase under mixing	Faster vesicle formation; simpler than film hydration in some setups	Sensitive to solvent ratio, injection rate, and mixing conditions	Can yield smaller vesicles; properties strongly influenced by solvent fraction and mixing regime	[12,19]
Sonication-assisted downsizing	Acoustic energy reduces vesicle size after initial vesicle formation	Convenient, effective for nanosizing	May induce local heating, leakage, or membrane perturbation	Usually decreases in size; may affect PDI, entrapment, and deformability depending on intensity/time	[12,19,20,41]
Membrane extrusion	Vesicles are forced through defined membrane pores	Better control of vesicle size and population uniformity	Additional processing step; outcome depends on pore size, cycles, and temperature	Narrows size distribution; improves size control; may alter apparent deformability	[12,19,38]
Homogenization/high-energy processing	Mechanical shear or pressure is applied to reduce vesicle size and homogenize dispersion	Potentially more scalable than simple lab methods	Equipment-dependent; may stress soft vesicles	Can improve uniformity and reduce size, but may also affect retention and membrane organization	[12,19]
Freeze-drying followed by reconstitution	Vesicles are dehydrated for storage and later reconstituted	Improves long-term stability when properly optimized	Requires cryo-/lyoprotectants; risk of fusion or leakage on reconstitution	May preserve vesicles for storage, but can change size and entrapment if not adequately protected	[29,31]

**Table 3:** Competing mechanistic interpretations of transferosome-mediated delivery.

Mechanistic Interpretation	Main Assumption	Supporting Rationale or Evidence	Main Limitation	References
Intact vesicle penetration	Transferosomes preserve vesicular integrity while adapting their shape and moving through narrow barrier-associated pathways	Classical transferosome theory links deformability, hydration gradients, and non-occlusive skin delivery to vesicle-mediated transport	Difficult to prove that intact vesicles cross the barrier deeply and remain structurally preserved throughout the process	[16,24]
Local drug release before or during barrier interaction	Transferosomes act mainly as reservoirs that retain the drug until contact with the barrier, followed by release and diffusion of the free drug	Enhanced delivery may occur even when the dominant event is drug release at or within the barrier rather than intact vesicle transport	Does not fully explain cases where vesicle composition, deformability, and barrier interaction strongly influence deposition or permeation	[14,16,77,78]
Barrier perturbation by edge activators	Surfactants or bile salts modify local lipid organization, interfacial tension, or barrier fluidity, thereby increasing drug partitioning or diffusion	Edge activator type and surface charge can significantly affect deformability and skin delivery behavior	May blur the distinction between vesicle-mediated delivery and classical penetration enhancement	[25,33]
Vesicle–barrier interfacial adaptation	Transferosomes improve delivery by establishing close contact with the barrier, promoting lipid exchange, local deposition, or superficial penetration	Consistent with evidence showing formulation-dependent changes in tissue deposition, permeation, and topical performance	Does not necessarily demonstrate complete trans-barrier transport of intact vesicles	[14,77,78]
Hybrid mechanism	Intact vesicle transport, partial penetration, local restructuring, surfactant-mediated barrier modulation, drug release, and subsequent diffusion may all contribute	Best accommodates the diversity of experimental outcomes reported for different drugs, vesicle compositions, barriers, and dosage forms	Requires formulation-specific mechanistic validation rather than reliance on a universal model	[14,15,16,25,33,61,77,78]

**Table 4:** Representative transferosome formulations and remaining unmet needs.

Drug or Active Compound	Intended Route/Application	Main Formulation Rationale	Reported Advantage	Remaining Unmet Need	References
Diclofenac	Topical/transdermal delivery	Ultradeformable vesicles designed to enhance skin delivery	Improved topical and transdermal delivery compared with conventional approaches	Need for clearer separation between intact vesicle transport, local drug release, and barrier perturbation	[16]
Asiatic acid	Topical anti-inflammatory delivery	Transferosomal gel designed to improve skin permeation and local delivery	Enhanced permeation and flux relative to non-transferosomal controls	Need for stronger correlation among edge activator selection, deformability, release behavior, and biological performance	[33]
Phloretin	Transdermal delivery	Transferosomal gel designed to improve skin penetration and systemic exposure	Increased skin penetration and improved in vivo pharmacokinetic performance	Need for broader validation across skin models, stability conditions, and clinically relevant comparators	[61]
Genistein	Topical melanoma-oriented delivery	Transferosome-embedded topical system for local skin delivery	Promising in vitro and ex vivo performance for melanoma-oriented topical application	Need for additional mechanistic, in vivo, and translational validation	[46]
Curcumin	Topical ocular delivery	Transferosomes designed to improve the ocular tissue penetration of a poorly soluble bioactive	Enhanced penetration into several ocular tissues	Need for further evaluation under clinically relevant ocular conditions and longer-term stability assessment	[21]
Tonabersat	Ocular delivery	Transferosomes designed for topical ocular administration	Improved penetration-related performance with acceptable physicochemical properties	Need for deeper assessment of tear-fluid effects, residence time, and clinical relevance	[51]
Resveratrol	Intranasal delivery	Nanosized transferosome-based in situ gel for brain targeting	Enhanced bioavailability and brain-targeting potential	Need for stronger evidence distinguishing nasal absorption, formulation residence, and true nose-to-brain contribution	[81]
Donepezil	Intranasal/nose-to-brain delivery	Hyaluronic-acid-doped nanotransfersomes for brain-targeted delivery	Improved delivery strategy for Alzheimer’s disease-oriented administration	Need for mechanistic confirmation, reproducibility, and translational validation	[82]
Loratadine	Buccal delivery	Buccal transferosomal gel designed to improve mucosal delivery	Improved ex vivo permeation and favorable pharmacokinetic performance in human volunteers	Need for broader clinical comparison and standardized mucosal performance testing	[84]
Fluconazole	Topical antifungal delivery	Transferosomal gel designed to enhance local antifungal activity	Improved antifungal formulation performance and compatibility	Need for longer-term stability, scalability, and standardized comparison with marketed topical products	[32]
Insulin	Transdermal macromolecular delivery	Transferosomal gel designed for non-invasive peptide delivery	Demonstrated feasibility of optimized transdermal insulin formulation	Need for stronger clinical feasibility data and robust evidence of macromolecular barrier transport	[35]

**Table 5:** Comparative features of transferosomes, liposomes, ethosomes, and transethosomes.

Feature	Liposomes	Transferosomes	Ethosomes	Transethosomes
Main composition	Phospholipids ± cholesterol [78]	Phospholipids + edge activator [15,19]	Phospholipids + high ethanol [77,85]	Phospholipids + ethanol + edge activator [15,86]
Key design principle	Vesicular encapsulation [78]	Membrane deformability [15,19]	Ethanol-assisted barrier interaction [77,85]	Combined ethanol effect + membrane deformability [15,86]
Typical membrane behavior	Comparatively more rigid/cohesive [78]	Ultradeformable, stress-adaptable [15,19]	Softened/fluidized by ethanol [77,85]	Highly flexible, hybrid behavior [15,86]
Dominant delivery rationale	Local deposition/reservoir effect [78]	Barrier adaptation and non-invasive transport [15,19]	Barrier lipid perturbation and enhanced partitioning [77,85,87]	Synergistic enhancement of penetration [15,86]
Main advantages	Biocompatibility; simple composition; versatile drug loading [78]	Strong interfacial adaptability; useful for dermal/transdermal delivery [15,19]	High permeation-promoting potential; useful for lipophilic drugs [77,85,87]	Strong penetration potential; combines two enhancement strategies [15,86]
Main limitations	Limited penetration across intact skin [78]	Reproducibility; stability; deformability standardization [19]	Ethanol-related stability/tolerability issues in some systems [77,85]	Greater formulation complexity; harder mechanistic interpretation [15,86]
When most useful	Superficial/local delivery [78]	When deformability and controlled barrier interaction are needed [15,19]	When ethanol-enhanced partitioning is advantageous [77,85,87]	When maximizing penetration justifies higher complexity [15,86]

## Data Availability

The original contributions presented in this study are included in the article. Further inquiries can be directed to the corresponding author.

## Abbreviations

The following abbreviations are used in this manuscript:

BBB	Blood–brain barrier
CQA	Critical quality attribute
QbD	Quality by Design
PDI	Polydispersity index
EE	Encapsulation efficiency
DLS	Dynamic light scattering
TEM	Transmission electron microscopy
cryo-TEM	Cryogenic transmission electron microscopy
AFM	Atomic force microscopy
DSC	Differential scanning calorimetry
FTIR	Fourier-transform infrared spectroscopy
XRD	X-ray diffraction
SAXS	Small-angle X-ray scattering
SANS	Small-angle neutron scattering
HPLC	High-performance liquid chromatography
UV–Vis	Ultraviolet–visible spectroscopy
SPC	Soy phosphatidylcholine
EPC	Egg phosphatidylcholine
DMPC	1,2-Dimyristoyl-sn-glycero-3-phosphocholine
DOE	Design of experiments
MIRIBEL	Minimum Information Reporting in Bio–Nano Experimental Literature

## References

[1] Yang R., Wei T., Goldberg H., Wang W., Cullion K., Kohane D.S. (2017). Getting Drugs Across Biological Barriers. Adv. Mater..

[2] Mitchell M.J., Billingsley M.M., Haley R.M., Wechsler M.E., Peppas N.A., Langer R. (2021). Engineering Precision Nanoparticles for Drug Delivery. Nat. Rev. Drug Discov..

[3] Zhao Z., Ukidve A., Kim J., Mitragotri S. (2020). Targeting Strategies for Tissue-Specific Drug Delivery. Cell.

[4] Mitragotri S. (2013). Devices for Overcoming Biological Barriers: The Use of Physical Forces to Disrupt the Barriers. Adv. Drug Deliv. Rev..

[5] Alkilani A., McCrudden M.T., Donnelly R. (2015). Transdermal Drug Delivery: Innovative Pharmaceutical Developments Based on Disruption of the Barrier Properties of the Stratum Corneum. Pharmaceutics.

[6] Ensign L.M., Cone R., Hanes J. (2012). Oral Drug Delivery with Polymeric Nanoparticles: The Gastrointestinal Mucus Barriers. Adv. Drug Deliv. Rev..

[7] Boegh M., Nielsen H.M. (2015). Mucus as a Barrier to Drug Delivery—Understanding and Mimicking the Barrier Properties. Basic Clin. Pharmacol. Toxicol..

[8] Cone R.A. (2009). Barrier Properties of Mucus. Adv. Drug Deliv. Rev..

[9] Wu D., Chen Q., Chen X., Han F., Chen Z., Wang Y. (2023). The Blood–Brain Barrier: Structure, Regulation and Drug Delivery. Sig. Transduct. Target. Ther..

[10] Daneman R., Prat A. (2015). The Blood–Brain Barrier. Cold Spring Harb. Perspect. Biol..

[11] Romero E., Morilla M. (2013). Highly Deformable and Highly Fluid Vesicles as Potential Drug Delivery Systems: Theoretical and Practical Considerations. Int. J. Nanomed..

[12] Fernández-García R., Lalatsa A., Statts L., Bolás-Fernández F., Ballesteros M.P., Serrano D.R. (2020). Transferosomes as Nanocarriers for Drugs across the Skin: Quality by Design from Lab to Industrial Scale. Int. J. Pharm..

[13] Cevc G. (1996). Transfersomes, Liposomes and Other Lipid Suspensions on the Skin: Permeation Enhancement, Vesicle Penetration, and Transdermal Drug Delivery. Crit. Rev. Ther. Drug Carr. Syst..

[14] Rai S., Pandey V., Rai G. (2017). Transfersomes as Versatile and Flexible Nano-Vesicular Carriers in Skin Cancer Therapy: The State of the Art. Nano Rev. Exp..

[15] Ascenso A., Batista C., Cardoso P., Mendes T., Praça F., Bentley V., Raposo S., Simões S. (2015). Development, Characterization, and Skin Delivery Studies of Related Ultradeformable Vesicles: Transfersomes, Ethosomes, and Transethosomes. Int. J. Nanomed..

[16] Cevc G., Blume G. (2001). New, Highly Efficient Formulation of Diclofenac for the Topical, Transdermal Administration in Ultradeformable Drug Carriers, Transfersomes. Biochim. Biophys. Acta (BBA)-Biomembr..

[17] Matharoo N., Mohd H., Michniak-Kohn B. (2024). Transferosomes as a Transdermal Drug Delivery System: Dermal Kinetics and Recent Developments. WIREs Nanomed. Nanobiotechnol.

[18] Jose Morilla M., Lilia Romero E. (2016). Carrier Deformability in Drug Delivery. Curr. Pharm. Des..

[19] Opatha S.A.T., Titapiwatanakun V., Chutoprapat R. (2020). Transfersomes: A Promising Nanoencapsulation Technique for Transdermal Drug Delivery. Pharmaceutics.

[20] Abdallah M.H., Abu Lila A.S., Shawky S.M., Almansour K., Alshammari F., Khafagy E.-S., Makram T.S. (2022). Experimental Design and Optimization of Nano-Transfersomal Gel to Enhance the Hypoglycemic Activity of Silymarin. Polymers.

[21] Barbalho G.N., Brugger S., Raab C., Lechner J.-S., Gratieri T., Keck C.M., Rupenthal I.D., Agarwal P. (2024). Development of Transferosomes for Topical Ocular Drug Delivery of Curcumin. Eur. J. Pharm. Biopharm..

[22] Karnam S., Jindal A.B., Paul A.T. (2024). Quality by Design-Based Optimization of Teriflunomide and Quercetin Combinational Topical Transferosomes for the Treatment of Rheumatoid Arthritis. Int. J. Pharm..

[23] Benson H.A. (2006). Transfersomes for Transdermal Drug Delivery. Expert Opin. Drug Deliv..

[24] Cevc G., Blume G. (1992). Lipid Vesicles Penetrate into Intact Skin Owing to the Transdermal Osmotic Gradients and Hydration Force. Biochim. Biophys. Acta (BBA) -Biomembr..

[25] El Zaafarany G.M., Awad G.A.S., Holayel S.M., Mortada N.D. (2010). Role of Edge Activators and Surface Charge in Developing Ultradeformable Vesicles with Enhanced Skin Delivery. Int. J. Pharm..

[26] Khan I., Needham R., Yousaf S., Houacine C., Islam Y., Bnyan R., Sadozai S.K., Elrayess M.A., Elhissi A. (2021). Impact of Phospholipids, Surfactants and Cholesterol Selection on the Performance of Transfersomes Vesicles Using Medical Nebulizers for Pulmonary Drug Delivery. J. Drug Deliv. Sci. Technol..

[27] Duangjit S., Opanasopit P., Rojanarata T., Ngawhirunpat T. (2013). Evaluation of Meloxicam-Loaded Cationic Transfersomes as Transdermal Drug Delivery Carriers. AAPS PharmSciTech.

[28] Moqejwa T., Marimuthu T., Kondiah P.P.D., Choonara Y.E. (2022). Development of Stable Nano-Sized Transfersomes as a Rectal Colloid for Enhanced Delivery of Cannabidiol. Pharmaceutics.

[29] Franzé S., Selmin F., Samaritani E., Minghetti P., Cilurzo F. (2018). Lyophilization of Liposomal Formulations: Still Necessary, Still Challenging. Pharmaceutics.

[30] Ingvarsson P.T., Yang M., Nielsen H.M., Rantanen J., Foged C. (2011). Stabilization of Liposomes during Drying. Expert Opin. Drug Deliv..

[31] Lu Y., Cheng B., Shan Y., Zhou S., Xu C., Fei Y., Pan J., Piao J., Li F., Zhu Z. (2024). Lyophilization Enhances the Stability of Panax Notoginseng Total Saponins-Loaded Transfersomes without Adverse Effects on Ex Vivo/In Vivo Skin Permeation. Int. J. Pharm..

[32] Cheng Z., Kandekar U., Ma X., Bhabad V., Pandit A., Liu L., Luo J., Munot N., Chorage T., Patil A. (2024). Optimizing Fluconazole-Embedded Transfersomal Gel for Enhanced Antifungal Activity and Compatibility Studies. Front. Pharmacol..

[33] Opatha S.A.T., Titapiwatanakun V., Boonpisutiinant K., Chutoprapat R. (2022). Preparation, Characterization and Permeation Study of Topical Gel Loaded with Transfersomes Containing Asiatic Acid. Molecules.

[34] Rasheed M.S., Ansari S.F., Shahzadi I. (2022). Formulation, Characterization of Glucosamine Loaded Transfersomes and In Vivo Evaluation Using Papain Induced Arthritis Model. Sci. Rep..

[35] Malakar J., Sen S.O., Nayak A.K., Sen K.K. (2012). Formulation, Optimization and Evaluation of Transferosomal Gel for Transdermal Insulin Delivery. Saudi Pharm. J..

[36] Long L., Zhang J., Yang Z., Guo Y., Hu X., Wang Y. (2020). Transdermal Delivery of Peptide and Protein Drugs: Strategies, Advantages and Disadvantages. J. Drug Deliv. Sci. Technol..

[37] Medhi J., Thalluri C., Vasam M., Bukke S.P.N. (2025). The Future of Vesicular Drug Delivery: Transferosomes in Therapeutic Advancement—Applications, Innovations and Challenges. BioMed. Eng. OnLine.

[38] Qushawy M., Nasr A., Abd-Alhaseeb M., Swidan S. (2018). Design, Optimization and Characterization of a Transfersomal Gel Using Miconazole Nitrate for the Treatment of Candida Skin Infections. Pharmaceutics.

[39] Ramkanth S., Anitha P., Gayathri R., Mohan S., Babu D. (2021). Formulation and Design Optimization of Nano-Transferosomes Using Pioglitazone and Eprosartan Mesylate for Concomitant Therapy against Diabetes and Hypertension. Eur. J. Pharm. Sci..

[40] Kharwade R., Ali N., Gangane P., Pawar K., More S., Iqbal M., Bhat A.R., AlAsmari A.F., Kaleem M. (2023). DOE-Assisted Formulation, Optimization, and Characterization of Tioconazole-Loaded Transferosomal Hydrogel for the Effective Treatment of Atopic Dermatitis: In Vitro and In Vivo Evaluation. Gels.

[41] Omar M.M., Hasan O.A., El Sisi A.M. (2019). Preparation and Optimization of Lidocaine Transferosomal Gel Containing Permeation Enhancers: A Promising Approach for Enhancement of Skin Permeation. Int. J. Nanomed..

[42] Abdelwahd A., Abdul Rasool B.K. (2022). Optimizing and Evaluating the Transdermal Permeation of Hydrocortisone Transfersomes Formulation Based on Digital Analysis of the In Vitro Drug Release and Ex Vivo Studies. Recent Adv. Drug Deliv. Formul..

[43] Öztürk K., Kaplan M., Çalış S. (2024). Effects of Nanoparticle Size, Shape, and Zeta Potential on Drug Delivery. Int. J. Pharm..

[44] Rodriguez-Loya J., Lerma M., Gardea-Torresdey J.L. (2023). Dynamic Light Scattering and Its Application to Control Nanoparticle Aggregation in Colloidal Systems: A Review. Micromachines.

[45] Mirza R., Shah K.U., Khan A.U., Fawad M., Rehman A.U., Ahmed N., Nawaz A., Shah S.U., Alasmari A.F., Alharbi M. (2024). Statistical Design and Optimization of Nano-Transfersomes Based Chitosan Gel for Transdermal Delivery of Cefepime. Drug Dev. Ind. Pharm..

[46] Motawea A., Maria S.N., Maria D.N., Jablonski M.M., Ibrahim M.M. (2024). Genistein Transfersome-Embedded Topical Delivery System for Skin Melanoma Treatment: In Vitro and Ex Vivo Evaluations. Drug Deliv..

[47] Hallan S.S., Sguizzato M., Esposito E., Cortesi R. (2021). Challenges in the Physical Characterization of Lipid Nanoparticles. Pharmaceutics.

[48] Bhattacharjee S. (2016). DLS and Zeta Potential—What They Are and What They Are Not?. J. Control. Release.

[49] Bhujbal S., Agarwal P., Sengupta S., Keck C.M., Rupenthal I.D. (2025). Influence of Tear Fluid Properties on Physicochemical, Mucoadhesion and Ocular Penetration Characteristics of Transfersomes. Eur. J. Pharm. Biopharm..

[50] Wang J.-P., Huang Z.-R., Zhang C., Ni Y.-R., Li B.-T., Wang Y., Wu J.-F. (2025). Methodological Advances in Liposomal Encapsulation Efficiency Determination: Systematic Review and Analysis. J. Drug Target..

[51] Bhujbal S., Rupenthal I.D., Agarwal P. (2025). Formulation and Characterization of Transfersomes for Ocular Delivery of Tonabersat. Pharm. Dev. Technol..

[52] Salem H., Attia S., Abdelmohsen H., Ali M. (2015). Preparation and Clinical Evaluation of Nano-Transferosomes for Treatment of Erectile Dysfunction. Drug Des. Dev. Ther..

[53] Cárdenas M., Campbell R.A., Yanez Arteta M., Lawrence M.J., Sebastiani F. (2023). Review of Structural Design Guiding the Development of Lipid Nanoparticles for Nucleic Acid Delivery. Curr. Opin. Colloid Interface Sci..

[54] Castañeda-Reyes E.D., Perea-Flores M.D.J., Davila-Ortiz G., Lee Y., Gonzalez De Mejia E. (2020). Development, Characterization and Use of Liposomes as Amphipathic Transporters of Bioactive Compounds for Melanoma Treatment and Reduction of Skin Inflammation: A Review. Int. J. Nanomed..

[55] Rahbari R., Francis L., Guy O.J., Sharma S., Von Ruhland C., Xia Z. (2023). Microneedle-Assisted Transfersomes as a Transdermal Delivery System for Aspirin. Pharmaceutics.

[56] Ruozi B., Tosi G., Forni F., Fresta M., Vandelli M.A. (2005). Atomic Force Microscopy and Photon Correlation Spectroscopy: Two Techniques for Rapid Characterization of Liposomes. Eur. J. Pharm. Sci..

[57] Agrawal S.S., Baliga V., Londhe V.Y. (2024). Liposomal Formulations: A Recent Update. Pharmaceutics.

[58] Wu L., Zhang J., Watanabe W. (2011). Physical and Chemical Stability of Drug Nanoparticles. Adv. Drug Deliv. Rev..

[59] Musakhanian J., Rodier J.-D., Dave M. (2022). Oxidative Stability in Lipid Formulations: A Review of the Mechanisms, Drivers, and Inhibitors of Oxidation. AAPS PharmSciTech.

[60] Ontong J.C., Singh S., Siriyong T., Voravuthikunchai S.P. (2024). Transferosomes Stabilized Hydrogel Incorporated Rhodomyrtone-Rich Extract from Rhodomyrtus Tomentosa Leaf Fortified with Phosphatidylcholine for the Management of Skin and Soft-Tissue Infections. Biotechnol. Lett..

[61] Wang J., Zhao Y., Zhai B., Cheng J., Sun J., Zhang X., Guo D. (2023). Phloretin Transfersomes for Transdermal Delivery: Design, Optimization, and In Vivo Evaluation. Molecules.

[62] Cevc G. (2012). Rational Design of New Product Candidates: The next Generation of Highly Deformable Bilayer Vesicles for Noninvasive, Targeted Therapy. J. Control. Release.

[63] Cevc G., Gebauer D. (2003). Hydration-Driven Transport of Deformable Lipid Vesicles through Fine Pores and the Skin Barrier. Biophys. J..

[64] Giordani S., Marassi V., Zattoni A., Roda B., Reschiglian P. (2023). Liposomes Characterization for Market Approval as Pharmaceutical Products: Analytical Methods, Guidelines and Standardized Protocols. J. Pharm. Biomed. Anal..

[65] Leong H.S., Butler K.S., Brinker C.J., Azzawi M., Conlan S., Dufés C., Owen A., Rannard S., Scott C., Chen C. (2019). On the Issue of Transparency and Reproducibility in Nanomedicine. Nat. Nanotechnol..

[66] Fan Y., Marioli M., Zhang K. (2021). Analytical Characterization of Liposomes and Other Lipid Nanoparticles for Drug Delivery. J. Pharm. Biomed. Anal..

[67] Mourdikoudis S., Pallares R.M., Thanh N.T.K. (2018). Characterization Techniques for Nanoparticles: Comparison and Complementarity upon Studying Nanoparticle Properties. Nanoscale.

[68] Faria M., Björnmalm M., Thurecht K.J., Kent S.J., Parton R.G., Kavallaris M., Johnston A.P.R., Gooding J.J., Corrie S.R., Boyd B.J. (2018). Minimum Information Reporting in Bio–Nano Experimental Literature. Nat. Nanotechnol..

[69] Thomas D.G., Klaessig F., Harper S.L., Fritts M., Hoover M.D., Gaheen S., Stokes T.H., Reznik-Zellen R., Freund E.T., Klemm J.D. (2011). Informatics and Standards for Nanomedicine Technology. WIREs Nanomed. Nanobiotechnol..

[70] Halamoda-Kenzaoui B., Vandebriel R.J., Howarth A., Siccardi M., David C.A.W., Liptrott N.J., Santin M., Borgos S.E., Bremer-Hoffmann S., Caputo F. (2021). Methodological Needs in the Quality and Safety Characterisation of Nanotechnology-Based Health Products: Priorities for Method Development and Standardisation. J. Control. Release.

[71] Zagalo D.M., Silva B.M.A., Silva C., Simões S., Sousa J.J. (2022). A Quality by Design (QbD) Approach in Pharmaceutical Development of Lipid-Based Nanosystems: A Systematic Review. J. Drug Deliv. Sci. Technol..

[72] Alshaer W., Nsairat H., Lafi Z., Hourani O.M., Al-Kadash A., Esawi E., Alkilany A.M. (2022). Quality by Design Approach in Liposomal Formulations: Robust Product Development. Molecules.

[73] Buya A.B., Mahlangu P., Witika B.A. (2024). From Lab to Industrial Development of Lipid Nanocarriers Using Quality by Design Approach. Int. J. Pharm. X.

[74] Lin H., Xie Q., Huang X., Ban J., Wang B., Wei X., Chen Y., Lu Z. (2018). Increased Skin Permeation Efficiency of Imperatorin via Charged Ultradeformable Lipid Vesicles for Transdermal Delivery. Int. J. Nanomed..

[75] Ghanbarzadeh S., Arami S. (2013). Enhanced Transdermal Delivery of Diclofenac Sodium via Conventional Liposomes, Ethosomes, and Transfersomes. BioMed Res. Int..

[76] Fathi-Azarbayjani A., Ng K.X., Chan Y.W., Chan S.Y. (2015). Lipid Vesicles for the Skin Delivery of Diclofenac: Cerosomes vs. Other Lipid Suspensions. Adv. Pharm. Bull..

[77] Natsheh H., Touitou E. (2020). Phospholipid Vesicles for Dermal/Transdermal and Nasal Administration of Active Molecules: The Effect of Surfactants and Alcohols on the Fluidity of Their Lipid Bilayers and Penetration Enhancement Properties. Molecules.

[78] Ashtikar M., Nagarsekar K., Fahr A. (2016). Transdermal Delivery from Liposomal Formulations—Evolution of the Technology over the Last Three Decades. J. Control. Release.

[79] El Maghraby G.M., Barry B.W., Williams A.C. (2008). Liposomes and Skin: From Drug Delivery to Model Membranes. Eur. J. Pharm. Sci..

[80] Ahmed S., Amin M.M., Sayed S. (2023). Ocular Drug Delivery: A Comprehensive Review. AAPS PharmSciTech.

[81] Salem H.F., Kharshoum R.M., Abou-Taleb H.A., Naguib D.M. (2019). Nanosized Transferosome-Based Intranasal In Situ Gel for Brain Targeting of Resveratrol: Formulation, Optimization, In Vitro Evaluation, and In Vivo Pharmacokinetic Study. AAPS PharmSciTech.

[82] Salem H.F., Aboud H.M., Abdellatif M.M., Abou-Taleb H.A. (2024). Nose-to-Brain Targeted Delivery of Donepezil Hydrochloride via Novel Hyaluronic Acid-Doped Nanotransfersomes for Alzheimer’s Disease Mitigation. J. Pharm. Sci..

[83] Aboud H.M., Ali A.A., El-Menshawe S.F., Elbary A.A. (2016). Nanotransfersomes of Carvedilol for Intranasal Delivery: Formulation, Characterization and In Vivo Evaluation. Drug Deliv..

[84] Elkomy M.H., El Menshawe S.F., Abou-Taleb H.A., Elkarmalawy M.H. (2017). Loratadine Bioavailability via Buccal Transferosomal Gel: Formulation, Statistical Optimization, In Vitro/In Vivo Characterization, and Pharmacokinetics in Human Volunteers. Drug Deliv..

[85] Almuqbil R.M., Aldhubiab B. (2025). Ethosome-Based Transdermal Drug Delivery: Its Structural Components, Preparation Techniques, and Therapeutic Applications Across Metabolic, Chronic, and Oncological Conditions. Pharmaceutics.

[86] Seenivasan R., Halagali P., Nayak D., Tippavajhala V.K. (2025). Transethosomes: A Comprehensive Review of Ultra-Deformable Vesicular Systems for Enhanced Transdermal Drug Delivery. AAPS PharmSciTech.

[87] Zhang J.-P., Wei Y.-H., Zhou Y., Li Y.-Q., Wu X.-A. (2012). Ethosomes, Binary Ethosomes and Transfersomes of Terbinafine Hydrochloride: A Comparative Study. Arch. Pharm. Res..

